# SI-ATRP Decoration of Magnetic Nanoparticles with PHEMA and Post-Polymerization Modification with Folic Acid for Tumor Cells’ Specific Targeting

**DOI:** 10.3390/ijms23010155

**Published:** 2021-12-23

**Authors:** Razvan Ghiarasim, Natalia Simionescu, Adina Coroaba, Cristina M. Uritu, Narcisa Laura Marangoci, Sorin-Alexandru Ibanescu, Mariana Pinteala

**Affiliations:** 1 Centre of Advanced Research in Bionanoconjugates and Biopolymers, “Petru Poni” Institute of Macromolecular Chemistry, 41A Grigore Ghica Voda Alley, 700487 Iasi, Romania; ghiarasim.razvan@icmpp.ro (R.G.); natalia.simionescu@icmpp.ro (N.S.); adina.coroaba@icmpp.ro (A.C.); nmarangoci@icmpp.ro (N.L.M.); 2 “Prof. Dr. Nicolae Oblu” Emergency Clinical Hospital, 2 Ateneului Street, 700309 Iasi, Romania; 3 Centre for Advanced Research and Development in Experimental Medicine (CEMEX), “Grigore T. Popa” University of Medicine and Pharmacy, 16 Universitatii Street, 700115 Iasi, Romania; cristina-mariana.uritu@umfiasi.ro

**Keywords:** magnetic nanoparticles, folate receptors, folic acid, PHEMA, SI-ATRP, tumor cell lines

## Abstract

Targeted nanocarriers could reach new levels of drug delivery, bringing new tools for personalized medicine. It is known that cancer cells overexpress folate receptors on the cell surface compared to healthy cells, which could be used to create new nanocarriers with specific targeting moiety. In addition, magnetic nanoparticles can be guided under the influence of an external magnetic field in different areas of the body, allowing their precise localization. The main purpose of this paper was to decorate the surface of magnetic nanoparticles with poly(2-hydroxyethyl methacrylate) (PHEMA) by surface-initiated atomic transfer radical polymerization (SI-ATRP) followed by covalent bonding of folic acid to side groups of the polymer to create a high specificity magnetic nanocarrier with increased internalization capacity in tumor cells. The biocompatibility of the nanocarriers was demonstrated by testing them on the NHDF cell line and folate-dependent internalization capacity was tested on three tumor cell lines: MCF-7, HeLa and HepG2. It has also been shown that a higher concentration of folic acid covalently bound to the polymer leads to a higher internalization in tumor cells compared to healthy cells. Last but not least, magnetic resonance imaging was used to highlight the magnetic properties of the functionalized nanoparticles obtained.

## 1. Introduction

After cardiovascular disease, cancer is the second largest cause of mortality, exerting a tremendous financial and social burden on society. There were 17.5 million cancer cases and 8.7 million deaths globally in 2015 [1,2], and according to the American Cancer Society, there are anticipated 1.9 million additional cases in 2021 [3]. Several classical and novel approaches [4] have been adopted in recent decades to handle cancer treatment problems, depending on the nature of tumor tissue and disease severity. Combining various strategies to create novel therapeutic methods has become increasingly important in the current context. In this regard, more and more targeted nanocarriers present high interest as potential option for personalized medicine, especially in cancer diagnosis and therapy. Furthermore, targeted nanocarriers can be modified based on the aim and used to target a specific moiety [5]. These approaches contribute to the reduction of chemotherapeutics’ severe side effects [6,7,8,9,10].

A well-studied transporter is based on magnetic nanoparticles (MNP), an interesting class of metal oxides able to be oriented and even accumulated into desired tissue by an external magnetic field with a strong attractiveness for in vivo applications [11,12]. Furthermore, MNPs can be coated, functionalized and loaded with therapeutics to endow targeting place and drug release capabilities. Nevertheless, the resulting systems may exhibit good colloidal stability, high biocompatibility, low cytotoxicity, non-immunogenicity, increased blood circulation time and high drug loading with tailored release in physiological conditions [13,14]. Moreover, MNPs can be used in cancer screening and diagnosis as contrast agents for magnetic resonance imaging (MRI). To date, the most studied contrast agents have been those based on Fe^3+^, especially superparamagnetic iron oxide nanoparticles (SPIONs), which have shown a marked increase in contrast in the transverse relaxation (T2) [15,16].

MNPs have been functionalized with various types of both molecular moieties and macromolecular compounds, without affecting their bulk characteristics. Particularly important is their functionalization with polymers that are able to provide specific properties depending on their type [17]. Four strategies may be used to design MNP hybrid materials: (i) physical adsorption of polymers on the surface of uncoated MNPs [18] and covalent grafting of organic material on inorganic nanomaterial’s surface by (ii) “grafting-to”, (iii) “grafting-from” or (iv) “grafting through” [19,20].

The “grafting-to” approach consists in reacting the reactive groups attached to MNP surfaces with the end functionality of (bio)polymers that can subsequently load desired molecules, such as pharmaceutics or enzymes [11,12,19,21,22,23,24,25,26]. The covalent binding of the polymer to the surface of the nanoparticles has an increased efficiency compared to its absorption on the surface [27].

The “grafting-from” strategy occurs when an initiator has been previously attached to the MNP surface, triggering in situ polymerization of a monomer [19,28,29], whereas the “grafting-through” strategy involves anchoring a polymerizable group on the MNP’s surface, allowing for the grafting and growth of (co)polymer on the MNP’s surface in the presence of an initiator and (co)monomers in solution [19,30].

To summarize, the application, mainly in the biomedical field, will dictate the nature and method of MNP’s coverage taking into consideration the “passive” or “active” targeting mechanism. In this context, the Enhanced Permeability and Retention (EPR) effect is responsible for “passive” nanomaterial targeting, whereas “active” nanomaterial targeting entails adequate functionalization of nanomaterials’ surfaces with specific molecules, such as antibodies, folic acid (FA), enzymes, and so on, that can recognize the surface of targeted cells [18].

It is important to note the growing interest for the use of the “grafting-from” strategy in the coating of inorganic nanoparticles with polymers [20]. This tendency is due to the possibility of high control over the molecular weight and molecular weight distribution ensured by the use of controlled radical polymerization techniques such as the atom transfer radical polymerization (ATRP) method. Additionally, such a strategy provides reversible terminated functionalities significant in controlling the final properties of the obtained polymers [28].

The application of this scenario involves the use of acrylic monomers readily polymerizable by ATRP. Consequently, one of the most common approaches uses N,N-dimethylaminoethyl methacrylate [31,32], oligo(ethylene glycol) methyl ether methacrylate [33], and poly(ethylene glycol) methacrylate [34] monomers for grafting MNPs.

Special interest has been recently focused on poly(2-hydroxyethyl methacrylate) (PHEMA) obtained by ATRP polymerization of 2-hydroxyethyl methacrylate (HEMA) monomer [35]. Due to its structure with a high concentration of hydroxyl groups attached to the main polymer chain, easily convertible to carboxylic groups, PHEMA may initiate cyclic esters’ ring-opening polymerization. It can also react with specific molecules, such as drugs and targeting molecules, to improve their bioactivity [36,37]. To add, it improves other properties, such as hydrophilicity (due to high density of hydroxyl groups) and biocompatibility by acting in a similar way as poly(ethylene glycol) (PEG) with its non-fouling properties over a wide range of grafting densities and molecular weights [38]. Furthermore, PHEMA brushes have been shown to keep their non-fouling properties, while allowing specific interactions after being modified with biological active molecules [39].

In terms of targeting molecules, FA is known to identify cells that overexpress the folate receptor on their surface [40]. FA has been used as a targeting molecule on a wide range of nanocarriers, such as polymer-coated magnetic nanoparticles, small copolymers loaded with active principles, liposomes, and hydrogels [41,42,43,44,45,46,47]. Furthermore, normal cells and malignant cells have distinct membrane chemical compositions and exhibit different surface properties, and this particularity can be exploited for the development of targeted nanoparticles [48,49,50,51].

The purpose of this paper is to present the results of a study aimed to binding FA onto the surface of MNPs via “grafting from” strategy, using the non-fouling PHEMA layer grown by surface-initiated ATRP (SI-ATRP) in order to create a magnetic nanocarrier with high specificity and high ability to internalize into cancer cells. Subsequently, PHEMA was functionalized with FA, and the biocompatibility of the resulted nanocarrier was investigated by testing on the NHDF cell line, as well as internalizing it through the folate receptor on three tumor cell lines: MCF-7, HeLa and HepG2. It has also been shown that a higher concentration of FA covalently linked to the polymer leads to a higher internalization on tumor cells compared to healthy cells. During the study, the properties of the obtained nanocarrier were monitored by MRI.

## 2. Results and Discussion

**Scheme 1:**
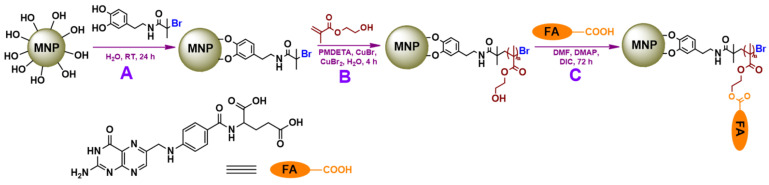
Synthesis steps for obtaining nanocarrier: A—grafting of the ATRP initiator on the surface of MNPs; B—HEMA polymerization by SI-ATRP; C—functionalization of PHEMA with FA. MNP—magnetic nanoparticle; FA—folic acid; RT—room temperature; PMDETA—N,N,N′,N′′,N′′-pentamethyldiethylenetriamine; DMF—dimethylformamide; DMAP—4-dimethylaminopyridine; DIC—N,N’-diisopropylcarbodiimide.

An inorganic nucleus (MNPs) coated with a layer of polymer later functionalized with FA (targeting molecule) was used to obtain the platform, fulfilling the targeting function at the cell surface, where it interacts with the folate receptor and leads to an internalization more efficient than simple MNPs. An overview of all the synthetic steps is presented in Scheme 1.

The unmodified MNPs were synthesized by the co-precipitation method and the main physicochemical analyses are presented and discussed in the Supplementary material (Figure S1). An ATRP polymerization initiator (Scheme S1), presenting a catechol anchor in its structure, was grafted on the surface of the unmodified MNPs, resulting in MNP-I nanoplatforms. The chemical structure of the initiator was evidenced by nuclear magnetic resonance spectroscopy (Figure S2) and is discussed in the Supplementary material. The HEMA monomer was subsequently polymerized on the surface of MNP-I nanoplatforms by using the SI-ATRP method. After the polymerization step, the hydroxyl groups of the side chains of PHEMA were reacted with FA via a N,N’-diisopropylcarbodiimide (DIC) catalyzed reaction. It was assumed that FA grafted onto nanocarriers represents an efficient targeting method for malignant cells, which overexpress folate receptors on their cell membranes [40]. Furthermore, we hypothesized that higher contents of FA in MNP-P-FA determine higher cellular uptake. To verify these assumptions, MNP-P-FA samples were synthesized with two concentrations of FA, denoted as MNP-P-FA1 and MNP-P-FA2.

### 2.1. Fourier-Transform Infrared Spectroscopy (FTIR)

**Figure 1:**
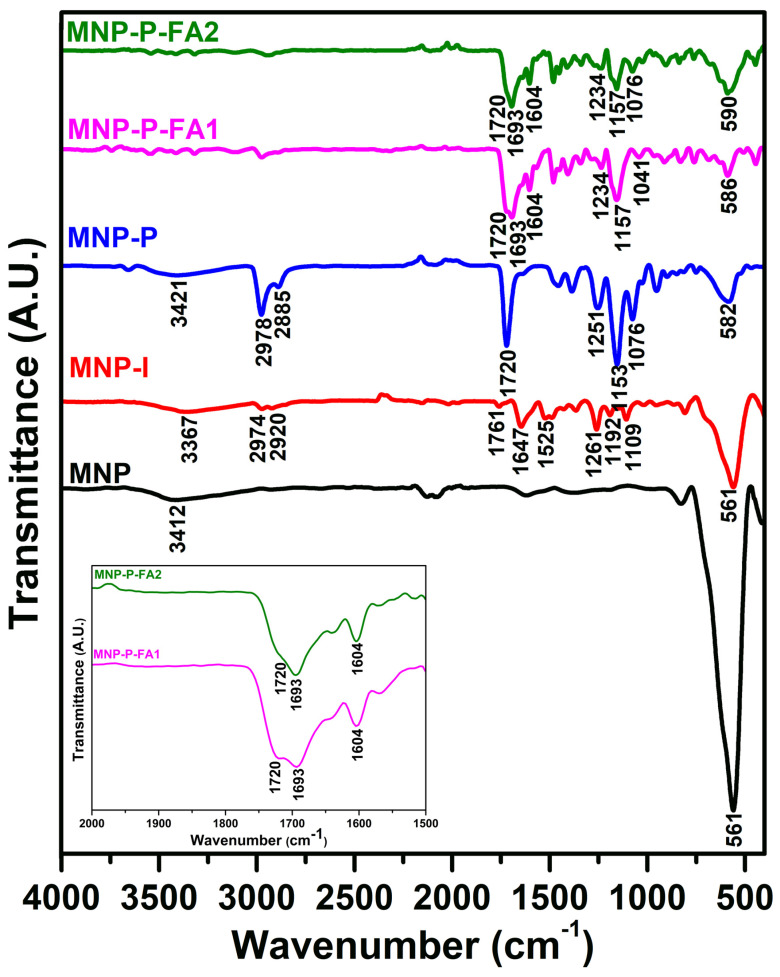
FTIR spectra of MNP (black), MNP-I (red), MNP-P (blue), MNP-P-FA1 (magenta), and MNP-P-FA2 (green). Insert represents the amplified area exhibited by the amide band of MNP-P-FA2 (green). A.U. – arbitrary units.

The FTIR spectrum of the uncoated MNPs (Figure 1. MNP) shows a vibration band at 561 cm^−1^ attributed to the Fe-O bond and at 3412 cm^−1^ characteristic to the OH group. Successful grafting of the ATRP initiator on the surface of the MNP is observed in the FTIR spectrum (Figure 1. MNP-I) by the appearance of a series of bands specific to the initiator, such as one from the 1109 cm^−1^ (C-H deformation band in the benzene nucleus), 1192 cm^−1^ (C-O vibration), 1261 cm^−1^ (C-C-C asymmetric stretching vibration), 1647 cm^−1^ (C=C vibration band in the benzene nucleus), 1761 cm^−1^ (C=O band), 1525 cm^−1^ (C-N band in the amide group), 2974 cm^−1^ (C-H vibration band from -CH_3_), 2920 cm^−1^ (C-H vibration band from -CH_2_-), 3367 cm^−1^ (N-H vibration band from the amide group), and 561 cm^−1^ characteristics of the vibration of Fe-O band. In the case of SI-ATRP of HEMA (Figure 1. MNP-P), there can be observed the appearance of the bands at 2978 and 2885 cm^−1^ characteristic of C-H vibration from the groups -CH_3_ and -CH_2_-, respectively, from the main polymer chain (PHEMA), the band from 3421 cm^−1^ OH vibration characteristic from the side chains of PHEMA, an intense band at 1720 cm^−1^ C=O bond characteristic for the PHEMA repetitive structural units, and the vibration bands at 1251, 1153 and 1076 cm^−1^ assigned to C-C, C-O, and C-O-C, respectively. Additionally, a vibration band at 582 cm^−1^ attributed to the Fe-O bond from MNP can be observed. The presence of the polymer on the surface of the MNPs is also demonstrated by the complete disappearance of the C=C vibration band at 1632 cm^−1^, which is specific to the HEMA vinyl monomer.

The functionalization of grafted PHEMA on the MNPs surface with FA in two different concentrations is highlighted by the appearance of two characteristic bands of FA in the FTIR spectrum (Figure 1. MNP-P-FA1 and MNP-P-FA2), such as the one from 1604 cm^−1^ attributed to the vibration band C=C of phenyl moiety and the one from 1653 cm^−1^ characteristic vibration band of the amide bond (insert in Figure 1) [52]. The Fe-O bond can be observed at 586 and 590 cm^−1^ for MNP-P-FA1 and MNP-P-FA2, respectively.

### 2.2. Ultraviolet-Visible Spectroscopy (UV-Vis)

**Figure 2:**
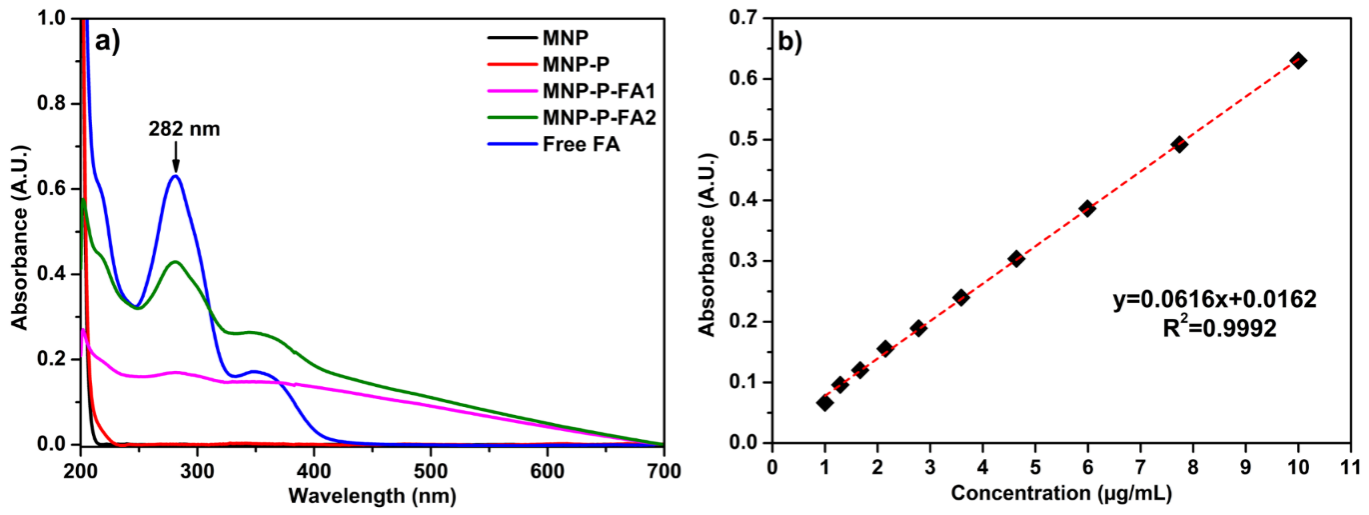
(**a**) UV-Vis spectra of MNP (black), MNP-P (red), MNP-P-FA1 (magenta), MNP-P-FA2 (green), and free FA (blue); (**b**) FA calibration curve at peak 282 nm with concentration 1 ÷ 10 μg/mL. A.U.—arbitrary units.

The UV-Vis spectra in Figure 2a, displaying the absorption curves of MNP, MNP-P, MNP-P-FA1, and MNP-P-FA2, allow quantification of the concentration of FA in their structure. First of all, the UV-vis spectra of MNP and MNP-P samples suggest that they do not absorb in the ultraviolet-visible range, while the nanocarriers with FA into their structures (MNP-P-FA1 and MNP-P-FA2) showed an absorption band at 282 nm, being similar to those of free FA. Using the calibration curve of FA in phosphate buffered saline (PBS) (Figure 2b and Figure S3), the FA concentration in the MNP-P-FA1 and MNP-P-FA2 nanocarriers was determined as 31.5 μg FA/mg MNP-P-FA1 and 83.5 μg FA/mg MNP-P-FA2.

### 2.3. X-ray Photoelectron Spectroscopy (XPS)

The surface chemistry of the synthesized MNP, MNP-I, MNP-P and MNP-P-FA1 was investigated by XPS (Figure S4). The presence of iron and oxygen was detected in the wide scan of uncoated MNP (Figure S4a), whereas nitrogen, carbon and bromide were recognized together with iron and oxygen in the wide scan of the sample containing the initiator (Figure S4b). Only oxygen, carbon and a small amount of nitrogen were identified by XPS analysis after the grafting of PHEMA on the surface of the MNPs (Figure S4c), whereas the surface examination of the sample containing FA (Figure S4d) confirmed the presence of the same three elements (oxygen, nitrogen and carbon).

**Figure 3:**
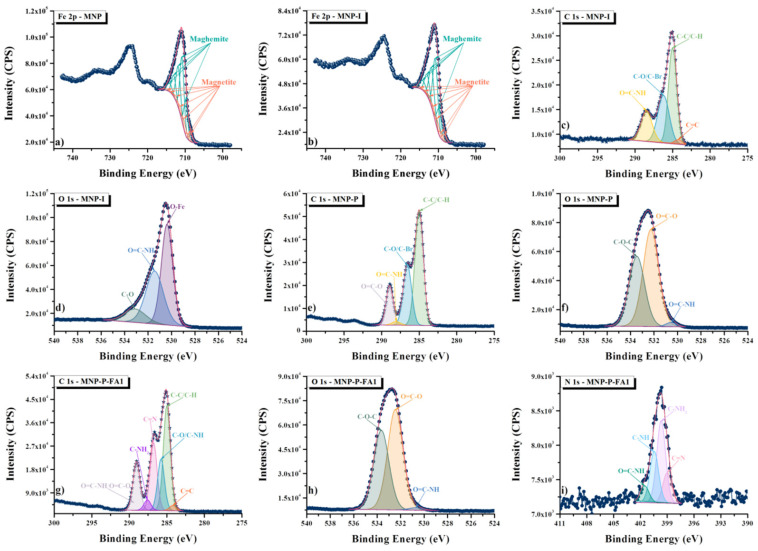
XPS high resolution spectra corresponding to Fe 2p for (**a**) MNP and (**b**) MNP-I, C 1s for (**c**) MNP-I, (**e**) MNP-P, and (**g**) MNP-P-FA1, O 1s for (**d**) MNP-I, (**f**) MNP-P and (**h**) MNP-P-FA1, and N 1s for (**i**) MNP-P-FA1. CPS – counts per second.

The oxidation state of iron at the outer surfaces was further investigated by high-resolution XPS of MNP (Figure 3a) and MNP-I (Figure 3b). The Fe 2p high-resolution spectra revealed the presence of mixed maghemite-magnetite nanoparticles, which were fitted using the theoretical multiplet peak created by Gupta and Sen [53] and as shown by Chowdhury and Yanful [54]. Theoretical multiplet analysis of the maghemite–magnetite mixed composition yielded 80.35% maghemite and 19.65% magnetite in the MNP sample (Figure 3a), whereas 80.10% maghemite and 19.90% magnetite was found in the nanoparticles coated with the initiator (Figure 3b). The C 1s deconvolution of the MNP-I sample allowed the identification of four peaks attributed to C=C, C-C/C-H, C-O/C-Br, and O=C-NH bonds (Figure 3c), mainly related to the synthesized initiator. The deconvolution of the O 1s signal also demonstrated the formation of the amidic bond (Figure 3d). The deconvolutions of C 1s and O 1s signals for the MNP-P sample are shown in Figure 3e,f. The appearance of one peak characteristic for the O=C-O bond, the “linker” between the initiator and the PHEMA layer, can be seen in both spectra. Despite the fact that XPS is a surface method with limited depth penetration (approximately 10 nm), the amidic bond (O=C-NH) was still visible after PHEMA grafting, indicating that the PHEMA layer is thinner than 10 nm. XPS also validated the functionalization of FA on the grafted PHEMA on the surface of MNP (MNP-P-FA1, Figure 3g–i). Six peaks were identified and assigned after deconvolution of the C 1s signal (Figure 3g): one peak assigned to C=C bond from the benzene ring of the FA, one peak assigned to C-C/C-H bonds from both FA and PHEMA, one peak assigned to C-O bond from PHEMA and C-NH bond from FA, one peak attributed to C=N bond, one peak attributed to C-NH_2_ bond from FA, and one peak assigned to O=C-NH/O=C-O bonds specific of both PHEMA and FA. Following the deconvolution of the O 1s signal (Figure 3h), only three peaks were identified, which were ascribed to O=C-NH, O=C-O, and C-O-C bonds, whereas the N 1s (Figure 3i) peak fitting revealed four peaks attributed to C=N, C-NH2, C-NH, and O=C-NH bonds.

### 2.4. Thermal Gravimetric (TGA) and Derivative Thermal Gravimetric (DTG) Analysis

**Figure 4:**
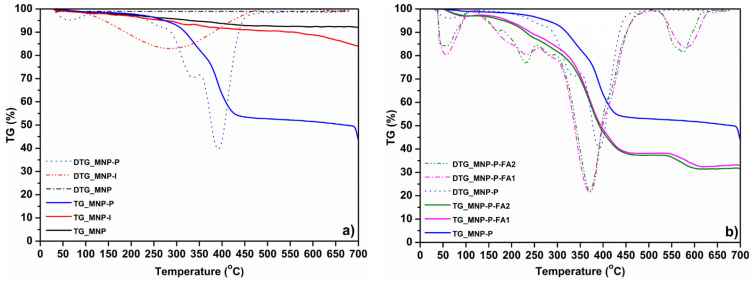
Thermal gravimetric/derivative thermal gravimetry (TGA/DTG) curves of: (**a**) MNP (black), MNP-I (red), MNP-P (blue), and (**b**) MNP-P (blue), MNP-P-FA1 (magenta), and MNP-P-FA2 (green).

Analyzing the thermogravimetric curves and derivative thermogravimetric curves in Figure 4, it is obvious that for the MNP and MNP-I samples (Figure 4a), no significant steps are observed. However, it can be observed that for MNP, a mass loss of 5.41% in 30 ÷ 490 °C temperature range, with a residual mass of 92.16%, does exist, while for the MNP-I sample, a mass loss of 2.16% can be observed due to water loss in the temperature range of 30 ÷ 160 °C, a loss of 5.04% in the range of 160 ÷ 480 °C, coming from the grafted ATRP initiator at T_max_ = 287 °C, and finally, a residual mass of 84.68% was obtained due to the inorganic residue. In the case of the MNP-P sample (Figure 4a), a degradation stage is observed in the range of 30 ÷ 160 °C, with a mass loss of 5.07% at T_max_ = 61 °C, due to water removal, which is followed by a significant degradation stage of polymer in 160 ÷ 490 °C temperature range with a mass loss of 39.78% at T_max_ = 391 °C, and finally, a residual mass of 44.02% was obtained, coming mainly from the inorganic residue of the nanocarrier. After the functionalization of MNP-P sample with the first FA concentration (MNP-P-FA1), the thermogravimetric analysis (Figure 4b) showed a first degradation stage in 40 ÷ 160 °C interval with a mass loss of 3.41% at T_max_ = 57 °C due to water loss, a second degradation stage in the temperature range of 160 ÷ 490 °C with a mass loss of 57.66% at T_max_ = 368 °C due to the organic material from the FA functionalized polymer. The degradation curve also showed a mass loss of 5.58% at T_max_ = 583 °C, in the range of 540 ÷ 612 °C, and finally remaining a residual mass of 32.75% due to the inorganic residue. In the case of the second concentration of FA (MNP-P-FA2), the TGA and DTG curves (Figure 4b) showed the first degradation stage of 3.96% in the range of 36 ÷ 160 °C with T_max_ = 53 °C due to water loss, the second stage of 58.54% in the range of 160 ÷ 490 °C with T_max_ = 371 °C due to the organic material, and the third degradation stage of 6.28% in the temperature range of 540 ÷ 612 °C at T_max_ = 576 °C and with a residual mass of 31.16%. If the thermal degradation curves (the second degradation stage corresponding to the organic material) of samples MNP-P-FA1 and MNP-P-FA2 (Figure 4b) are compared, a slight increase in mass loss is observed, which may represent that MNP-P-FA2 has a higher content of FA, and both samples are characterized by a third loss stage which can be due to the volatilization of the decomposed organic material in the incipient stages of degradation [55]. TGA curves allowed us to estimate the degree of polymerization of grafted PHEMA on the surfaces of MNP. The TGA losses for the organic material of MNP-I and MNP-P samples were permitted to estimate the degree of PHEMA polymerization of around 35 (M_w(PHEMA_) ≈ 4550 Da) (Supplementary material). Moreover, from the same curves, the number of grafted molecules per unit of inorganic area (nm^2^) was estimated using Equation (1) from the Supplementary Material [56]. As a result, the grafting density of the ATRP initiator on MNP’s surface was around 1.18 molecules/nm^2^ and of PHEMA was around 0.72 chains/nm^2^, resulting in a ratio of initiator molecules/PHEMA chains of 1.63. The slide difference between the numbers of initiator and PHEMA grafted on MNP’s surface could be due to secondary polymerization reactions or detachment of the initiator from the MNP’s surface [56].

The AAS method permitted the determination of iron contents from MNP, MNP-P, MNP-P-FA1 and MNP-P-FA2 samples as being 49.62%, 17.87%, 8.33% and 8.28%, respectively.

### 2.5. Differential Scanning Calorimetry (DSC)

**Figure 5:**
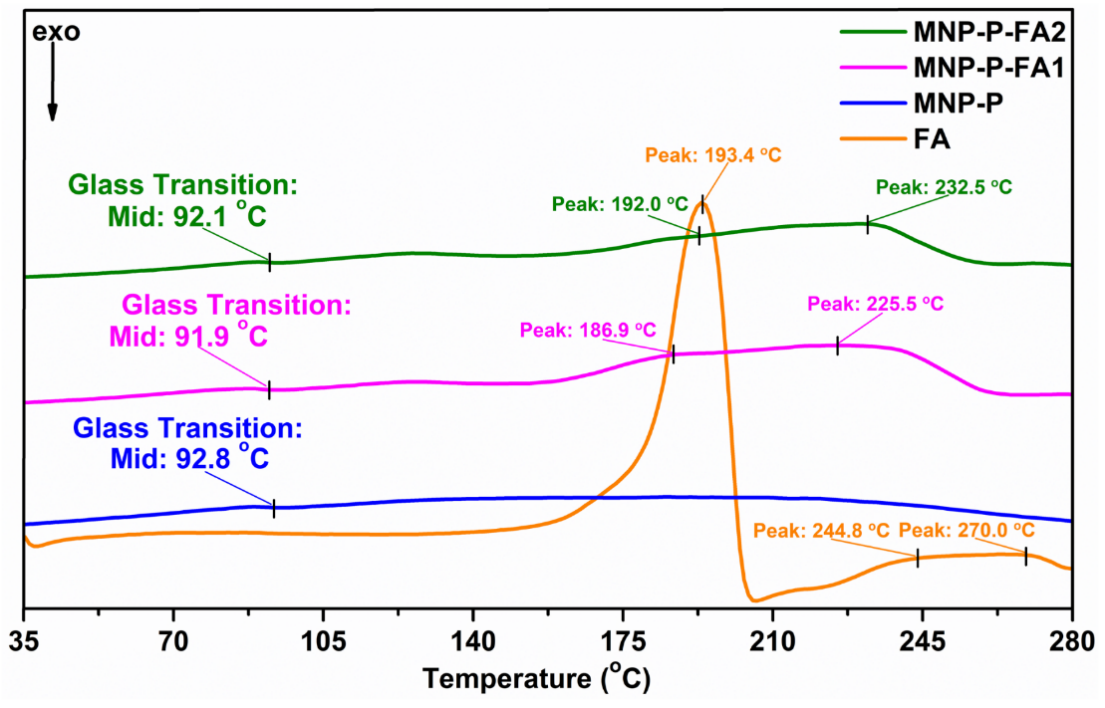
DSC curves of the studied samples: FA (orange), MNP-P (blue), MNP-P-FA1 (magenta), and MNP-P-FA2 (green). exo—exotherm.

DSC is an indispensable analytical tool in monitoring different solid state interactions. The registering of the heat flow versus temperature generates composition-dependent transitions in various types of materials, not limited to polymers [57,58,59,60]. Figure 5 shows the DSC curves of the studied structure and its comprising components. In the literature, PHEMA is presented to have a glass transition temperature domain (*Tg*) centered at 83 °C [61], but when PHEMA is grafted onto the MNPs’ surface, the *Tg* shifts with 10 °C (≈93 °C). This is due to a packing phenomenon, narrowing free volume between segmental chains and hindering their movement. The covalent linking of FA seems not to affect the *Tg* of PHEMA; instead, coupling FA to PHEMA together with the FA concentration causes changes in FA thermal behavior. The FA exhibits a sharp endothermic peak at 193 °C, attributed to the loss of glutamic acid moiety, and a low melting profile (245 °C), which overlaps with the onset of thermal degradation (270 °C) [62], but when FA is coupled to the PHEMA chain (MNP-P-FA1), the glutamic acid moiety is thermally protected, and the peak at 193 °C significantly decreases and shifts towards a lower temperature (187 °C). Furthermore, it coalesces with the peak of thermal degradation onset, which also shifts towards a lower temperature (226 °C), and the FA melting peak at 245 °C disappears, indicating a highly packed nanocluster [63].

With the increasing of FA concentration in the nanocluster (MNP-P-FA2), the double peak thermal degradation profile of MNP-P-FA1 at 186.9 and 225.5 °C shifts slightly towards 192.0 and 232.5 °C, respectively, making us conclude that the thermal stability of the nanocluster is influenced by FA’s concentration into the composition of nanocarriers.

### 2.6. Magnetic Properties (VSM)

**Figure 6:**
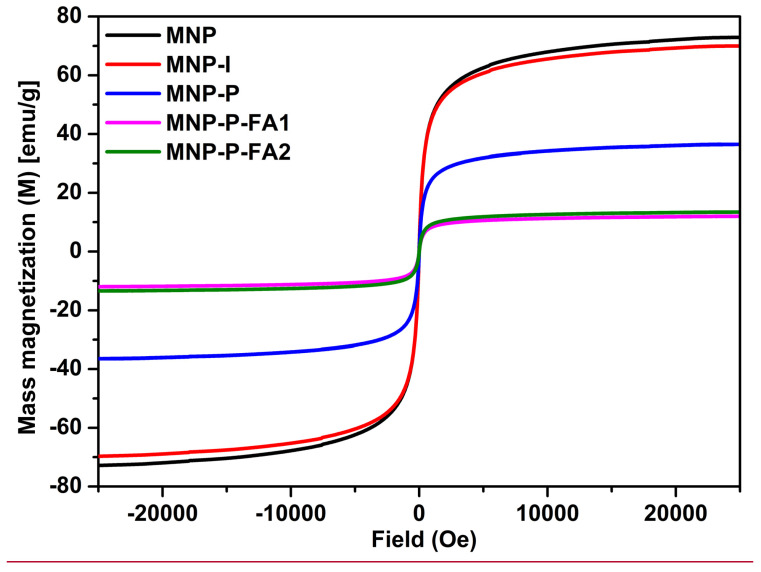
VSM magnetization at 2.5 T of MNP (black), MNP-I (red), MNP-P (blue), MNP-P-FA1 (magenta) and MNP-P-FA2 (green).

Magnetization measurements of MNP, MNP-I, MNP-P, MNP-P-FA1 and MNP-P-FA2 samples by VSM at 2.5 T are presented in Figure 6. First of all, as it can be observed from Figure 6, the magnetization curve of the MNP sample presents a lack of hysteresis, denoting that the analyzed sample has a superparamagnetic behavior with a saturation magnetization of 72.86 emu/g, confirming the fact that the method used to synthesize MNP produced superparamagnetic nanoparticles. When the surfaces of MNPs were modified, the saturation magnetization values dropped to 69.60, 36.47, 11.97 and 13.40 emu/g, corresponding to MNP-I, MNP-P, MNP-P-FA1 and MNP-P-FA2 samples, respectively, and, also, their superparamagnetic behavior is preserved, since there is no coercivity or remanence [64].

The grafting of the initiator onto the surface of MNP induced only a small difference in magnetization value but a significant decrease in the value of the saturation magnetization was observed after SI-ATRP polymerization of HEMA monomer on the MNP-I’s surface. Moreover, the saturation magnetization values decreased even more after the covalent linkages of FA to the grafted PHEMA on MNP-I’s surface. It should be highlighted that the difference in saturation magnetization values between MNP and MNP-P or MNP-P-FA is a metric that indicates the presence of a high density of polymer chains on the surface of MNP, resulting in a decrease in the amount of magnetic saturation. However, a saturation magnetization value of 12–14 emu/g or times larger is often recognized for biological applications [65]. Additionally, in our case, it should be noted that the increased concentration of FA at the surface of MNP-P nanocarriers did not dramatically influence the values of the saturation magnetization.

### 2.7. Dynamic Light Scattering (DLS) and Zeta Potential

**Figure 7:**
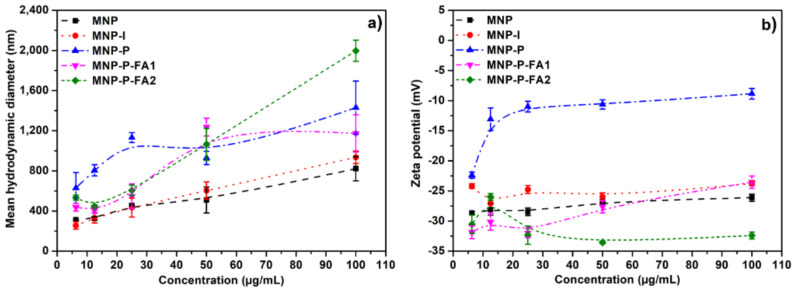
Hydrodynamic diameter (**a**) and Zeta potential (**b**) of MNP (black), MNP-I (red), MNP-P (blue), MNP-P-FA1 (magenta) and MNP-P-FA2 (green).

Figure 7a and Table S1 present the hydrodynamic diameter depending on the concentration in PBS of the studied samples (MNP, MNP-I, MNP-P, MNP-P-FA1 and MNP-P-FA2). From this figure, it can be observed that once the concentrations increase, the values of the hydrodynamic diameters increase, leading us to the idea that there is a tendency to agglomerate as the concentration increases. Additionally, the samples have the smallest hydrodynamic diameters when the concentration is less than 50 µg/mL, when the nanocarriers tend to be dispersed, and they may be employed in biological tests. This information must be completed with the surface charge of nanocarriers, giving information about colloidal stability, which governs the blood circulation, biodistribution, tumor accumulation and penetration, as well as their intracellular trafficking [66,67]. Thus, negatively charged nanoparticles exhibit lower retention in the blood and higher tissue accumulation compared to positively charged particles [66].

The surface charge was determined by the Zeta potential (ξ) in PBS at the same concentration variation as in the case of determining the hydrodynamic diameters (Figure 7b and Table S1). Thus, unmodified MNPs presented a ξ value of −26.12 ± 0.60 mV (Table S1) at a concentration of 100 μg/mL, with an increase in ξ value (−28.67 ± 0.23 mV) as the concentration decreases at 6.25 μg/mL, indicating that this type of nanoparticles has moderate colloidal stability in PBS. Their decoration with the ATRP initiator (MNP-I) leads to a decrease in colloidal stability (ξ = −23.77 ± 0.36 mV) and it is not dependent on sample concentration. Decreased colloidal stability can be observed in polymer-decorated nanoparticles (MNP-P), when, at a concentration of 100 μg/mL they have a ξ value of –8.86 ± 0.89 mV, with a slight improvement in their stability at 12.5 μg/mL (ξ = –13.09 ± 1.87 mV), followed by a sudden increased stability to –22.38 ± 0.51 mV at a concentration of 6.25 μg/mL when the nanocarriers exist in aggregates with the smallest hydrodinamic diameter (Figure 7a and Table S1). In the case of the first concentration of FA used in the functionalization of the polymer (MNP-P-FA1), ξ of –32.00 ± 0.74 mV is observed at a concentration of 25 μg/mL, remaining constant below the value of –30 mV (Table S1), along with decreasing in its concentration, indicating that this system has high colloidal stability. The MNP-P-FA2 sample shows a high colloidal stability over the entire concentration range except for the concentration of 12.5 μg/mL with a ξ value of –25.99 ± 0.55 mV, which indicates moderate colloidal stability [67], but once the MNP-P-FA2 concentration is increased the ξ value decreases (around −35 mV), concluding that a higher concentration of FA used in the functionalization of the polymer leads to increased colloidal stability of the whole nanocarrier.

### 2.8. Transmission Electron Microscopy (TEM) and Scanning Transmission Electron Microscopy (STEM)

As may be seen in the TEM images (Figure S5), a slight agglomeration tendency of MNP and MNP-I nanocarriers can be observed. However, they present spherical shapes with a narrow distribution with mean diameters of 8.91 and 9.56 nm, respectively, making them good candidates for subsequent functionalization.

**Figure 8:**
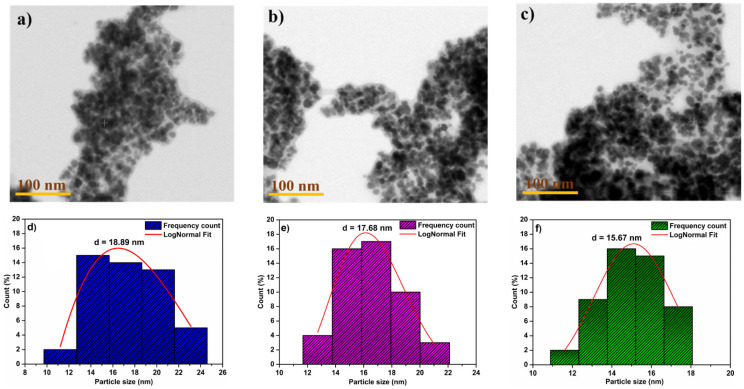
STEM images: (**a**) MNP-P; (**b**) MNP-P-FA1; (**c**) MNP-P-FA2 and histograms of particle size distribution: (**d**) MNP-P; (**e**) MNP-P-FA1; (**f**) MNP-P-FA2.

Because of the small dimensions of MNP-P, MNP-P-FA1 and MNP-P-FA2 nanocarriers and their agglomeration propensity, which was worsened by the drying process used to prepare TEM grids, instead of TEM, the STEM technique was utilized to highlight nanocarriers’ morphologies (Figure 8). MNP-P, MNP-P-FA1 and MNP-P-FA2 samples presented core-shell spherical structures (Figure 8a–c) with average diameters of 18.89, 17.68 and 15.67 nm, respectively (Figure 8d–f). These findings lead to two main conclusions: first, the presence of FA increases the individual stability of nanocarriers, and second, as the concentration of FA increases, the individual size of the nanocarrier decreases.

### 2.9. Biocompatibility of Nanocarriers

The nanocarrier was tested for biocompatibility on the NHDF (Normal Human Dermal Fibroblasts) cell line, continued by the study of nanocarrier internalization in both the NHDF cell line and a series of tumor cell lines (MCF-7 (breast adenocarcinoma), HeLa (cervical adenocarcinoma) and HepG2 (hepatocellular carcinoma)), ultimately leading to a determination of the internalization efficacy of the nanocarrier.

**Figure 9:**
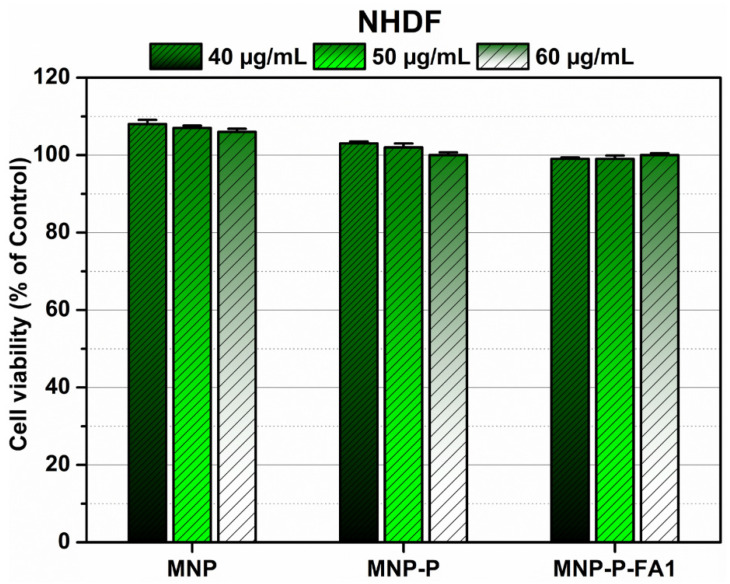
Biocompatibility of MNP, MNP-P and MNP-P-FA1 (40, 50 and 60 µg/mL) on normal human dermal fibroblasts (NHDF) after 24 h. Experiments were performed in triplicate, and treated cell viability was expressed as percentage of control cells’ viability. Data were represented as means ± standard error of the mean.

The biocompatibility of MNP, MNP-P and MNP-P-FA1 was determined by MTS assay after 24 h incubation with different sample concentrations (40, 50 and 60 µg/mL). The results showed that MNP, MNP-P and MNP-P-FA1 are biocompatible, as they do not affect normal fibroblasts viability (Figure 9). The nanocarriers are not cytotoxic against MCF-7, HeLa and HepG2 cancer cell lines (Figure S6).

### 2.10. Cellular Uptake of Nanocarriers

#### 2.10.1. Intracellular Iron Content by Thiocyanate Method

Cellular uptake of MNP, MNP-P and MNP-P-FA1 in NHDF, MCF-7, HeLa and HepG2 cells was estimated as iron uptake using the formula: $\%=\frac{Intracellular\;iron}{Incubated\;iron}\times 100$, where intracellular iron represents the iron measured in each well (with the thiocyanate method) and incubated iron represents total iron used at incubation/well. Iron uptake was then expressed as fold change of values obtained for MNP incubation, representing the cellular uptake of nanocarriers normalized to MNP uptake.

**Figure 10:**
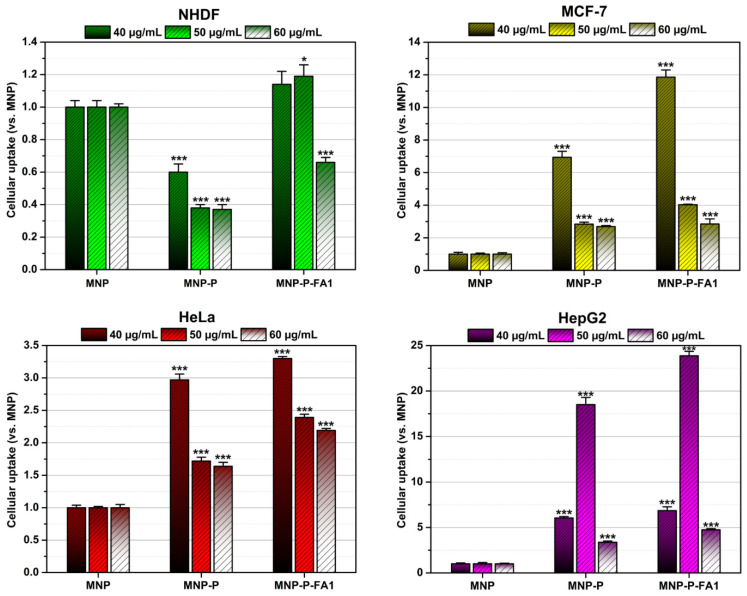
Cellular uptake of MNP, MNP-P and MNP-P-FA1 (40, 50 and 60 µg/mL) in normal human dermal fibroblasts (NHDF), breast adenocarcinoma (MCF-7), cervical adenocarcinoma (HeLa) and hepatocellular carcinoma (HepG2) cells after 24 h. Experiments were performed in triplicate; iron contents were determined with the thiocyanate method, and nanocarrier uptake was expressed as fold change of values obtained for MNP incubation. * *p* < 0.05, *** *p* < 0.001 vs. MNP (for each concentration, Student’s *t*-test).

PHEMA has non-fouling properties [68], suggesting that it should discourage cell–nanoparticle interactions that translate into cellular uptake. This is supported by our results in the case of NHDF cells, but not for cancer cells (Figure 10). This could be explained by differences in cell surface properties between normal and malignant cells, including cell surface potential. Although most cells have negative membrane potential [51], there have been conflicting reports regarding malignant cell surface charge. Abercrombie and Ambrose [51] showed that the cell surface of cancer cells presents higher negative charge compared to normal cells, partially explaining metastasis through reduced adhesiveness of tumor cells. However, Marikovsky et al. [69] showed that normal cells have a more pronounced negative cell surface charge compared to transformed cells. More recently, Zhang et al. [70] showed that MCF-10A normal cells have a more negative Zeta potential compared to MCF-7 cancer cells and that incubation with iron oxide nanoparticles determined opposite effects in the two cell lines, determined by binding and internalization effects. Thus, the Zeta potential value of MCF-10A cells increased slightly over 24 h incubation with iron oxide nanoparticles, possibly due to the internalization of nanoparticles, while the Zeta potential value of MCF-7 cells decreased significantly over 24 h incubation, possibly due to nanoparticle binding to the cell [70]. It has been shown that HeLa cells exhibit preferential cellular uptake of positively charged particles (PEG liposomes [71] and hydrogel nanoparticles [72]) compared to neutral or negatively charged particles. However, our results showed that functionalized MNPs with increasing negative Zeta potential (MNP-P > MNP-P-FA1) are internalized better than MNPs.

Furthermore, the results showed that MNP-P-FA1 cellular uptake was higher in MCF-7, HeLa and HepG2 cells compared to MNP or MNP-P uptake, but in NHDF cells, there was only a slight difference in uptake compared to MNP (Figure 10), suggesting that MNP-P-FA1 uptake is more efficient in malignant cells due to the overexpression of folate receptors on their cell membranes [40]. An interesting observation is that the cellular uptake of MNP-P-FA1 was inversely correlated with the concentrations used in the experiment. Thus, higher MNP-P-FA1 concentrations (i.e., 60 µg/mL) exhibit lower uptake, especially in MCF-7 and HeLa cells, probably due to nanocarriers’ aggregation in the cell culture medium, leading to large aggregates which are difficult to internalize. HepG2 showed the highest cellular uptake of MNP-P-FA1, but the obtained results did not follow the same trend as for the other cell lines and were independent of concentration, which could be due to the specific nature of their cell growth and metabolism.

#### 2.10.2. Intracellular Iron Content by Atomic Absorption Spectroscopy (AAS)

The cellular uptake of nanocarriers in NHDF and MCF-7 cells was estimated using intracellular iron contents determined by AAS. As determined by the thiocyanate method, MCF-7 presented the best results for cellular uptake out of the three tested malignant cell lines; therefore, it was chosen for subsequent experiments. Moreover, in these experiments, intracellular iron concentrations were determined using AAS, which is a much more sensitive method compared to the thiocyanate method, and it can be used to detect very low concentrations of iron. Cellular uptake of nanocarriers was calculated as above, intracellular iron in each well was reported to its respective protein content and normalized to MNP uptake.

**Figure 11:**
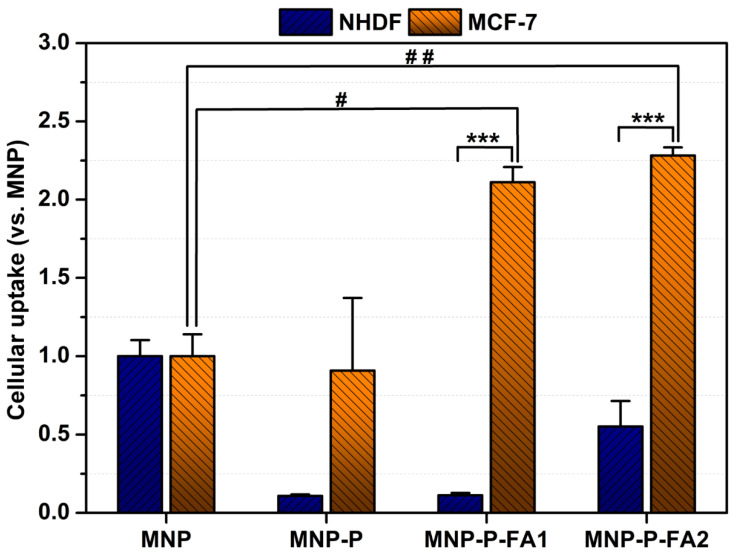
Cellular uptake of MNP, MNP-P, MNP-P-FA1 and MNP-P-FA2 (40 µg/mL) in normal human dermal fibroblasts (NHDF) and breast adenocarcinoma (MCF-7) cells after 24 h. Experiments were performed in triplicate, intracellular iron in each well was normalized to its respective protein content, and cellular uptake of nanocarriers was expressed as fold change of MNP uptake. *** *p* < 0.001 vs. NHDF cells (Student’s *t*-test); # *p* < 0.05, ## *p* < 0.01 vs. MNP (one-way ANOVA).

The obtained data confirmed that MNP-P-FA is internalized better in MCF-7 cells compared to NHDF cells (Figure 11). Furthermore, the increase in FA content in the nanocarriers determined a slight increase in the cellular uptake of MNP-P-FA2 compared to MNP-P-FA1 (Figure 11).

### 2.11. MRI of cells after incubation with nanocarriers

The production of MRI signal relies on the ability of certain atomic nuclei to absorb radio frequency (RF) energy when placed in an external magnetic field; the resultant spin polarization will induce a RF signal in a radiofrequency coil and consequently be detected [73].

**Figure 12:**
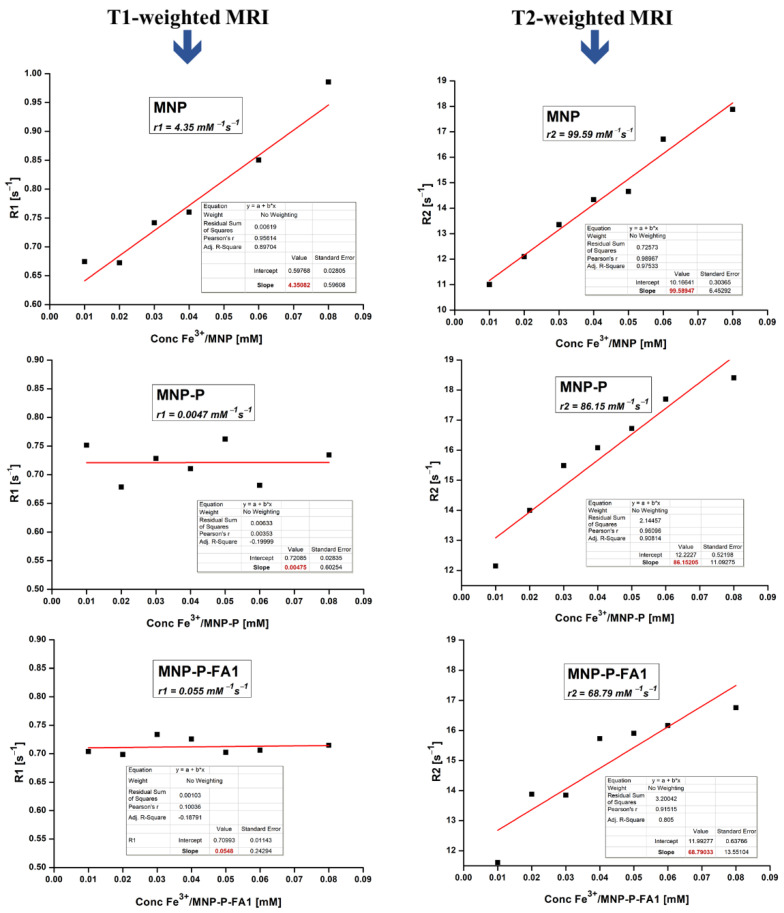
Linear fitting of 1/T1 (left column) and 1/T2 (right column) for MNP (first line), MNP-P (middle line) and MNP-P-FA1 (bottom line) as a function of [Fe^3+^].

MRI was utilized to assess the magnetic behavior of the shell of MNP used as a contrast agent. PBS solutions (pH 7.4) with different concentrations of MNP, MNP-P and MNP-P-FA1 samples were dispersed in 1% agar gel and were used for relaxivity MRI studies. By plotting the R1 and R2 as a function of samples concentration (as in Section 3.10), the *r1* and *r2* relaxivities were obtained, as illustrated in Figure 12. It could be noted that the values of *r1*, obtained in T1-weighted imaging, are very small, with a maximum of 4.35 mM^−1^s^−1^ for MNP, due to the fact that Fe^3+^ has proven poor MRI contrast features, as reported in a previous publication [74]. In contrast, the *r2* values, achieved in T2-weighted analysis, are significantly higher, ranging from 99.59 mM^−1^s^−1^ for MNP, very close to the relaxivity obtained for MNP-P of 86.15 mM^−1^s^−1^, and of 68.79 mM^−1^s^−1^ for MNP-P-FA1.

**Figure 13:**
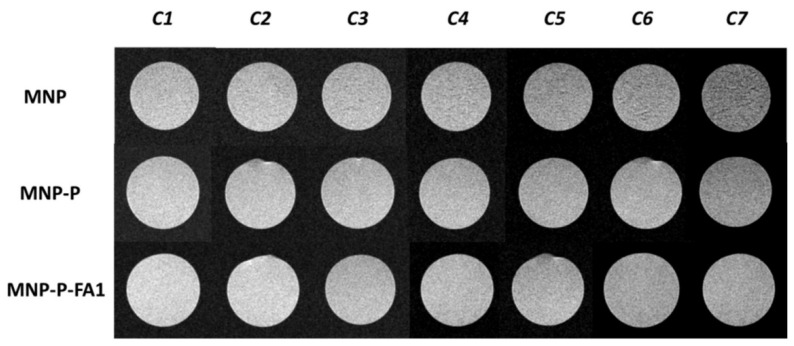
T2-weighted MR images of MNP, MNP-P and MNP-P-FA1 on the concentration range of Fe^3+^ between 0.1 and 0.8 mM (C1–C7) showing a negative contrast by increasing the concentration.

It is obvious that increasing the Fe^3+^ concentration results in shortening T2, and the images become darker (Figure 13). It should be mentioned that previous research has revealed that relaxivity is affected by the nature and the thickness of the polymeric coating of MNP [75]. In our case, a slight decrease of *r2* and *r2*/*r1* ratio in MNP-P-FA1 sample compared to that of MNP-P sample could be explained through the higher hydrodynamic diameter of MNP-P nanocarriers, which is consistent with previously reported findings [76].

## 3. Materials and Methods

### 3.1. Materials

Iron (II) chloride tetrahydrate (FeCl_2 ×_ 4H_2_O; 99%, Acros Organics, Geel, Belgium), iron (III) chloride hexahydrate (FeCl_3_x6H2O; Reag. Ph. Eur., >99%, Honeywell Fluka, Charlotte, NC, USA), ammonium hydroxide solution (NH_4_OH 25% in water; Honeywell Fluka, Charlotte, NC, USA), 3-hydroxytyramine hydrocloride (L-dopamine, 99%, Acros Organics, Geel, Belgium), α-bromoisobutyryl bromine (98%, Sigma-Aldrich, St. Louis, MO, USA), di-sodium tetraborate decahydrate (Na_2_B_4_O_7_x10H_2_O, Supelco, Bellefonte, PA, USA), sodium carbonate decahydrate (≥99.0%, Sigma-Aldrich, St. Louis, MO, USA), hydrochloric acid (36%, Supelco, Bellefonte, PA, USA), methanol (≥99.8%, Sigma-Aldrich, St. Louis, MO, USA), chloroform (99.0–99.4% (GC), Honeywell Fluka, Charlotte, NC, USA), 2-hydroxyethyl methacrylate (97%, HEMA, Sigma-Aldrich, St. Louis, MO, USA), N,N,N″,N″-pentamethyldiethylemetriamine (99%, PMDETA, Sigma-Aldrich, St. Louis, MO, USA), copper (I) bromine (≥98.0%, Honeywell Fluka, Charlotte, NC, USA), copper (II) bromine (99%, Sigma-Aldrich St. Louis, MO, USA), folic acid (≥97%, FA, Sigma-Aldrich, St. Louis, MO, USA), 4-(dimethylamino)pyridine (≥99.0%, DMAP Sigma-Aldrich, St. Louis, MO, USA), N-N’-diisopropylcarbodiimide (DIC, 99%, Sigma-Aldrich, St. Louis, MO, USA), N,N’-dimethylformamide pure (Lach-Ner, Neratovice, Czech Republic), phosphate buffered saline (PBS, 10X, Sigma-Aldrich, St. Louis, MO, USA). Normal human dermal fibroblasts (NHDF, PromoCell, Heidelberg, Germany), breast adenocarcinoma (MCF-7), cervical adenocarcinoma (HeLa) and hepatocellular carcinoma (HepG2) cells (all malignant cell lines purchased from CLS Cell Lines Service GmbH, Eppelheim, Germany) were grown in alpha-MEM medium (Lonza, Basel, Switzerland) supplemented with 10% fetal bovine serum (FBS, Gibco, Thermo Fisher Scientific, Waltham, MA, USA) and 1% Penicillin–Streptomycin–Amphotericin B mixture (10 K/10 K/25 μg, Lonza, Basel, Switzerland). All materials were used as received except for PHEMA, which was passed through an alumina column to remove the inhibitor and stored under inert atmosphere at −20 °C prior to polymerization).

### 3.2. Synthesis of Magnetic Nanoparticles (MNP)

The MNPs were obtained using the co-precipitation method, following a slightly modified literature protocol [11]. Briefly, 1.72 g of iron (II) chloride tetrahydrate (FeCl_2_x4H_2_O) and 4.70 g of iron (III) chloride hexahydrate (FeCl_3_x6H_2_O) were dissolved in 50 mL deionized water; then, the mixture was subjected to strong mechanical stirring under the inert atmosphere of N_2_ gas, at 80 °C, for 30 min. Afterward, 10 mL NH_4_OH (25% by weight NH_3_ in water) were added under vigorous stirring and N_2_ flow, until the pH reached 11, at which point a black suspension emerged; the stirring was continued for 30 min. The prepared nanoparticles were washed five times with purified water to remove excess NH_4_OH and lower the pH to 7. Subsequently, these nanoparticles were lyophilized and stored under an inert atmosphere.

### 3.3. Synthesis of Initiator for Surface-Initiated Atom Transfer Radical Polymerization (SI-ATRP Initiator)

The initiator of SI-ATRP with a chatecol anchor was synthesized following an adapted literature protocol [77]. Borax (Na_2_B_4_O_7_x10H_2_O, 3.83 g, 10 mmol) and 100 mL deionized water were added into a 250 mL round-bottomed flask. After degassing the solution by bubbling N_2_ gas for 30 min, dopamine hydrocloride (1.9 g, 10 mmol) was added. The pH of the reaction mixture was changed to pH 9–10 with Na_2_CO_3_x10H_2_O (3.99 g, 32 mmol) after 15 min of stirring. The resulting solution was cooled on an ice bath and then α-bromobutyryl bromine (1.236 mL, 2.29 g, 10 mmol) was added dropwise with a syringe. The reaction mixture was allowed to cool to room temperature under N_2_ gas and stirred for 24 h. During the reaction, the pH of the solution was maintained at 9–10 by adding Na_2_CO_3_xH_2_O. After 24 h, the reaction solution was acidified to pH 2 with 1 M aqueous HCl solution. Ethyl acetate was used to perform a liquid–liquid extraction of the organic compound. Organic fractions were dried over MgSO_4_, subsequently decreasing the solvent under reduced pressure and obtaining a brown viscous liquid. The purification of the obtained brown liquid was performed in two steps: firstly on a silica gel column (4% methanol in chloroform), resulting in a colorless viscous liquid, followed by crystallization from a 50:50 water:methanol solution, yielding white crystals (0.56 g, 2.6 mmol, yield 26%) that were stored under an inert atmosphere at −20 °C.

### 3.4. Decoration of Nanoparticles’ Surface with the Synthesized ATRP Initiator (MNP-I)

A total of 500 mg dry MNPs were dispersed by sonication for 15 min in 150 mL deionized water and then, the previously synthesized SI-ATRP initiator suspension (0.5 g, 2.3 mmol) was added. The suspension was mechanically stirred for 24 h, and then the modified MNPs were purified by multiple successive centrifugation/redispersion steps in deionized water to remove the unlinked initiator from the surface. The resulting initiator-modified nanoparticles were lyophilized and stored under an inert atmosphere at 4 °C.

### 3.5. Surface-Initiated Atom Transfer Radical Polymerization (MNP-P)

In a 25 mL flask, 4 mL HEMA monomer (1.073 g/mL, 4.292 g, 32 mmol), 344.51 μL PMDETA (0.83 g/mL, 0.285 g, 1.64 mmol), CuBr_2_ (0.0366 g, 0.16 mmol) and 4 mL deionized water were added and N_2_ gas was bubbled for 45 min. After this time, CuBr (0.0828 g, 0.57 mmol) was added to the solution under nitrogen flow and then bubbled for an additional 15 min. Afterward, under an inert atmosphere, the monomer solution was transferred over deoxygenated 8 mL water solution of 100 mg MNP-I. The final suspension was subjected to mechanical agitation, under light-protected conditions and an inert atmosphere for 4 h. The newly modified nanoparticles were lyophilized and stored under an inert atmosphere at 4 °C.

### 3.6. PHEMA Functionalization with FA (MNP-P-FA1 and MNP-P-FA2)

A total of 20 mg of MNP-P were dispersed in 10 mL dimethylformamide (DMF) in a 25 mL vial; then, 40 mg of FA (0.09 mmol) and 2.4 mg of DMAP (0.02 mmol) were added. After that, N_2_ gas was bubbled for one hour over the obtained dispersion. On an ice bath and inert atmosphere, 49.6 μL of DIC (0.040 g, 0.31 mmol) were added dropwise to the obtained solution. The suspension was allowed to cool to ambient temperature before being mechanically stirred for 48 h in an inert medium. Then, after centrifugation, the nanoparticles were washed three times with DMF to remove any unreacted FA, and the newly modified nanoparticles (MNP-P-FA1) were washed three times with methanol, followed by removing the solvent under reduced pressure at 40 °C. Respecting the same reaction conditions but by using 20 mg of MNP-P, 10 mL of DMF, 60 mg of FA (0.13 mmol), 3.6 mg of DMAP (0.0036 g, 0.47 mmol), and 74.4 μL DIC (0.060 g, 0.47 mmol), MNP-P-FA2 were obtained. The newly modified nanoparticles were stored under an inert atmosphere at 4 °C.

### 3.7. Characterization

The Raman spectrum was recorded using a inVia confocal microscope (Renishaw, Sheffield, UK) equipped with a He-Ne laser at 632.8 nm (17 mW) and a CCD detector coupled to a Leica DM 2500 M microscope. The measurements were performed in backscattering geometry using a 50× objective. The Raman measurements were performed at room temperature and atmospheric pressure.

For the analysis of samples by FTIR, a IRTracer-100 FTIR Spectrometer (Shimadzu, Kyoto, Japan) was used, involving the attenuated total reflection (ATR) mode with 36 scans in the spectral range 4000 ÷ 400 cm^−1^.

On a KRATOS Axis Nova (Kratos Analytical, Manchester, UK) with AlK radiation, 20 mA current, 15 kV voltage (300 W), and a base pressure of 10^−8^ to 10^−9^ Torr in the sample chamber, XPS was performed. The XPS survey spectra of the samples were obtained in the range of −5 to 1200 eV with a resolution of 1 eV and a pass energy of 160 eV. High-resolution spectra for all the elements identified from the survey spectra were collected using a pass energy of 20 eV and a step size of 0.1 eV. The C–C/C–H bond has been normalized at a binding energy of 285 eV, which was further used to calibrate the data. The analysis was performed using the ESCApe software.

A Lambda 35 apparatus (Perkin Elmer Inc., Waltham, MA, USA) was used to make UV-Vis measurements. Samples based on MNPs were analyzed at a concentration of 80 μg/mL in PBS 1X at pH 7.4. The FA calibration curve was also performed in PBS 1X at pH 7.4 at different concentrations of FA.

STA 449 F1 JUPITER (Netzsch, Selb, Germany) equipment were used to perform thermoanalytical measurements. Indium was used to perform temperature and sensitivity calibrations in the temperature range of 30 ÷ 700 °C. The temperature range for measurements was 30 to 700 °C, with a heating rate of 10 °C min^−1^ and a flow rate of 50 mL min^−1^ under a dry nitrogen environment. The NETZSCH PROTEUNS 4.2 software (Netzsch, Selb, Germany) was used to process the data.

The DSC measurements were conducted on a DSC 200 F3 Maia apparatus (Netzsch, Selb, Germany). A total of 5 mg of each sample was weighed into aluminum crucibles, afterward sealed shut with pierced lids. Nitrogen was used as an inert atmosphere (50 mL min^−1^ flow rate) at a heating rate of 10 °C min^–1^.

The hydrodynamic diameter of the nanoparticles was determined by DLS using a Delsa Nano C Submicron Particle Size Analyzer (Beckman Coulter, Brea, CA, USA) at concentrations of 100, 50, 25 and 12.5 μg/mL of sample in PBS 1X at pH 7.4. The samples were analyzed in triplicate with 70 iterations each, and the standard deviations were calculated.

The Zeta potential was measured using a Delsa Nano C Submicron Particle Size Analyzer (Beckman Coulter, Brea, CA, USA) using the corresponding module (Flow Cell Module). The samples were analyzed at concentrations of 100, 50, 25, 12.5 and 50 μg/mL of sample in PBS 1X at pH 7.4. The Zeta potential was measured at five points with 10 iterations in triplicate, and the standard deviations were calculated.

The morphological analysis of the unmodified nanoparticles and decorated with ATRP initiator was performed using TEM, involving a HT7700 Transmission Electron Microscope (Hitachi High-Tech Corporation, Tokyo, Japan) operated at an acceleration voltage of 100 kV in High-Contrast Mode. Samples at a concentration of 1 μg/mL PBS 1X pH 7.4 were dropwise deposited on carbon-coated copper grids having a 400-mesh size; then, the solvent was removed at reduced pressure. The ImageJ program was used to represent the size distribution of nanoparticles.

The morphology of the samples was investigated with a Scanning Electron Microscope type Verios G4 UC (Thermo Fisher Scientific, Waltham, MA, USA) working in STEM mode at 30 kV, with a STEM 3+ detector (Bright-Field Mode). The samples were prepared on carbon-coated copper grids with 300-mesh size. MNP-P, MNP-P-FA1 and MNP-P-FA2 dispersed in PBS 1X pH 7.4 (1 μg/mL) were placed on the grids, and then the solvent was removed under vacuum.

The saturation magnetizations of the nanoparticles were measured at 25 °C on a 8600 Series VSM (LakeShore Cryotronics, Westerville, OH, USA). Demagnetization was performed at 20 kOe and the magnetic properties were recorded up to a maximum field of 25 kOe (2.5 Tesla).

X-ray diffraction analysis was performed on a Miniflex 600 diffractometer (Rigaku, Tokyo, Japan) that uses CuKα emission in the angular range of 10 ÷ 80° (2θ) with a scan step of 0.01° and a recording rate of 1°/min.

The amount of iron in nanoparticles was determined by AAS. Nanoparticle suspensions (1 mg/mL) were digested overnight with 6 N HCl (1:4) at 60 °C on a shaker, then diluted and used for iron quantification at 248.327 nm using a ContrAA 800 high resolution atomic absorption flame spectrometer (Analytic Jena AG, Jena, Germany). Iron contents of nanoparticles were determined against a calibration curve (0 ÷ 5 μg/mL) and were expressed as μg Fe/mg nanocarrier.

### 3.8. Cytotoxicity Assay (MTS Assay)

The biocompatibility of MNP, MNP-P and MNP-P-FA was determined by MTS assay using the CellTiter 96^®^ Aqueous One Solution Cell Proliferation Assay (Promega, Madison, WI, USA), according to the manufacturers’ instructions. Cells were seeded into 96-well tissue culture-treated plates at a density of 0.6 × 10^5^ and allowed to adhere overnight. Cells were then incubated for 24 h with fresh complete medium (control) or different sample concentrations (40, 50 and 60 µg/mL). MTS reagent was added to each well 3 h prior to absorbance readings at 490 nm on a FLUOstar^®^ Omega microplate reader (BMG LABTECH, Ortenberg, Germany). Experiments were performed in triplicate and treated cells’ viability was expressed as a percentage of control cells’ viability.

### 3.9. Intracellular Iron Quantification

Intracellular iron concentrations were measured in order to estimate cellular uptake of nanocarriers, using two methods: thiocyanate method [78] and AAS.

#### 3.9.1. Thiocyanate Method

The quantification of intracellular iron in cells incubated with MNP, MNP-P and MNP-P-FA1 was performed using an adapted thiocyanate method [78]. Cells were seeded into 96-well tissue culture-treated plates at a density of 0.6 × 10^5^ cells/mL (NHDF) or 1.2 × 10^5^ cells/mL (MCF-7, HeLa, HepG2), allowed to adhere overnight, and then incubated for 24 h with fresh complete medium (control) or different sample concentrations (40, 50 and 60 µg/mL). After incubation, cells were washed twice with PBS and lysed with 100 µL 1 N HCl for 2 h at 60 °C on a shaker. Next, 10 µL ammonium persulfate (2 mg/mL) and 100 µL potassium thiocyanate (0.1 M) were added, the plate was shaken for 30 s, and the absorbance was read at 480 nm on a FLUOstar^®^ Omega microplate reader (BMG LABTECH, Ortenberg, Germany). Experiments were performed in triplicate, and iron concentrations were determined against a calibration curve. Nanocarriers’ cellular uptake was estimated as iron uptake using the formula: $\%=\frac{Intracellular\;iron}{Incubated\;iron}\times 100$, where intracellular iron represents the iron measured in each well and incubated iron represents total iron used at incubation/well. Iron uptake was then expressed as fold change of values obtained for MNP incubation, representing the cellular uptake of nanocarriers normalized to MNP uptake.

#### 3.9.2. Atomic Absorption Spectroscopy

Iron quantification by AAS was performed using a previously published method [79], with some modifications. Cells were seeded into 24-well tissue culture-treated plates at a density of 0.6 × 10^5^ cells/mL (NHDF) or 1.2 × 10^5^ cells/mL (MCF-7), allowed to adhere overnight, then incubated for 24 h with fresh complete medium (control) or medium containing 40 µg/mL MNP, MNP-P, MNP-P-FA1 or MNP-P-FA2. After 24 h, the medium was removed, and cells were washed with PBS and lysed with 200 µL 50 mM NaOH for 2 h at room temperature on a shaker. Cell lysates were collected and sonicated for 15 min; then, aliquots were used for the quantification of total iron by AAS and for the quantification of total cellular protein using the bicinchoninic acid assay. Total iron quantification by AAS was performed at 248.327 nm using a ContrAA 800 high resolution atomic absorption spectrometer with a graphite oven (Analytic Jena AG, Jena, Germany) and iron concentrations were determined against a calibration curve (0 ÷ 20 ng/mL). Nanocarriers’ cellular uptake was estimated as iron uptake using the formula: $\%=\frac{Intracellular\;iron}{Incubated\;iron}\times 100$, where intracellular iron represents the iron measured in each well, normalized to its respective protein content, and incubated iron represents total iron used at incubation/well. Iron uptake was then expressed as fold change of values obtained for MNP incubation, representing the cellular uptake of nanocarriers normalized to MNP uptake.

### 3.10. MRI Investigations

MNP, MNP-P and MNP-P-FA1 compounds were evaluated as MRI contrast agents using nanoScan PET-MRI instrument for small animals of 1 Tesla (Mediso LTD, Budapest, Hungary). A good MRI signal may afford to confirm the targeting properties of MNP-P-FA1, which is in direct connection with longitudinal (T1) or transversal (T2) relaxation times. With the help of the Nucline software of the instrument, the longitudinal and transversal relaxivities, *r1* and *r2*, respectively, were calculated as the slope of the linear regression of R1 (1/T1) and R2 (1/T2) on the range of sample concentrations, similar to a previous study we have published [80,81]. The samples were prepared using the corresponding dispersions in PBS (pH 7.4) of MNP, MNP-P and MNP-P-FA1, by adding the calculated volume in 1% agarose (at 45 °C) to gain the concentration of Fe^3+^ in the range of 0.01 ÷ 0.08 mM. Each sample was scanned in T1 and T2 mode, being placed in the center of the transmission/receiver coil, having B0 magnetic field shimming and coil calibration at water proton frequency. To determine the T1 relaxation times, 2D gradient echo (T1 GRE) sequences were recorded, having the following acquisition parameters: TR = 74 ms, TE = 3.8 ms, NSA = 2, slice thickness = 5 mm, in plane resolution = 0.2 mm. The T1 values for each concentration were calculated through a two-point estimation method (flip angles of 10 and 60°), using the following formula [82]:

(1) $$\ln\left[\frac{\left(I_{1}\sin\theta_{2}-I_{2}\sin\theta_{1}\right)}{\left(I_{1}\sin\theta_{2}\cos\theta_{1}-I_{1}\sin\theta_{2}\cos\theta_{1}\right)}\right]=\frac{-\mathrm{TR}}{T_{1}}$$

where: I_1_, I_2_—the mean signal intensities measured inside circular regions of interest (ROI) of 10 mm diameter, drawn inside the samples at 10 and 60° flip angles; TR—repetition time; *θ*_1_ and *θ*_2_—the flip angles.

The T2 relaxation times were obtained from a 2D spin echo sequence (T2 SE), with the acquisition parameters as follows: TR = 400 ms, TE = 20, 40, 80, 120 ms, NSA = 2, slice thickness = 5 mm, using the equation:

(2) $$I=A\cdot e^{\frac{TE}{T2}}$$

where: I—mean signal intensity in the ROI, A—initial intensity of the signal, TE—echo time.

### 3.11. Statistical Analysis

Statistical analysis was performed using GraphPad Prism 8 software (GraphPad Software Inc., San Diego, CA, USA). Data were expressed as means ± standard error of the mean and analyzed with one-way ANOVA with Fisher’s LSD test or independent two-tailed (Student’s) *t*-test, considering *p* < 0.05 statistically significant.

## 4. Conclusions

MNPs decorated with PHEMA of around 4550 Da with spherical nano-sizes and suitable for subsequent functionalization with FA were obtained by the SI-ATRP method. Using two different concentrations of FA in the functionalization of MNP-P, biocompatible nanocarriers with specific targeting capability were obtained. Nanocarriers with higher FA concentrations (MNP-P-FA2) had higher internalization efficiency in MCF-7 cells compared to normal cells and to nanocarriers with lower FA concentrations (MNP-P-FA1), suggesting that the FA concentration used in the functionalization has a significant influence on the targeting efficiency of the nanocarrier. The surface modifications of MNP with PHEMA did not decrease the *r2* relaxivity value significantly, but the linkages of FA to PHEMA decreased the *r2* value, being indirectly correlated with the hydrodynamic diameters of nanocarriers. Therefore, we can infer that the presence of Fe_3_O_4_ may enhance the contrast only in T2-weighted MR imaging, by a darker signal.

## Data Availability

The data presented in this study are available on request from the corresponding author.

## Supplementary Materials

The following are available online at www.mdpi.com/article/10.3390/ijms23010155/s1.
