# Supplementary material for: SI-ATRP Decoration of Magnetic Nanoparticles with PHEMA and Post-Polymerization Modification with Folic Acid for Tumor Cells’ Specific Targeting
Source: Int J Mol Sci. 2021 Dec 23;23(1):155. doi: 10.3390/ijms23010155 (PMC8745432; doi:10.3390/ijms23010155)
Supplement: Supplementary file 1 [file ijms-23-00155-s001.zip › ijms-1518073-supplementary.pdf]

Supplementary material

SI-ATRP decoration of magnetic nanoparticles with PHEMA and post-polymerization modification with folic acid for tumor cells' specific targeting

Razvan Ghiarasim [1, †], Natalia Simionescu [1, 2, †], Adina Coroaba [1], Cristina M. Uritu [3], Narcisa Laura Marangoci [1], Sorin-Alexandru Ibanescu [1, *] and Mariana Pinteala [1, *]

[1] Centre of Advanced Research in Bionanoconjugates and Biopolymers, "Petru Poni" Institute of Macromolecular Chemistry, Romanian Academy, 41A Grigore Ghica Voda Alley, 700487 Iasi, Romania; ghiarasim.razvan@icmpp.ro (R.G.); natalia.simionescu@icmpp.ro (N.S.); adina.coroaba@icmpp.ro (A.C.); nmarangoci@icmpp.ro (N.L.M.); ibanescu.sorin@icmpp.ro (S.A.I.); pinteala@icmpp.ro (M.P.)

[2] Emergency Clinical Hospital "Prof. Dr. Nicolae Oblu", 2 Ateneului Street, 700309 Iasi, Romania;

[3] Centre for Advanced Research and Development in Experimental Medicine (CEMEX), "Grigore T. Popa" University of Medicine and Pharmacy, 16 Universitatii Street, 700115 Iasi, Romania; cristina-mariana.uritu@umfiasi.ro (C.M.U.)

* Correspondence: ibanescu.sorin@icmpp.ro (S.A.I.); pinteala@icmpp.ro (M.P.); Tel.: +40-332-880-050 (M.P.)

† These authors contributed equally to this work.

Characterization of synthesized magnetic nanoparticles (MNP)

MNPs were synthesized by the method of co-precipitation of iron chlorides (II and III) in a basic medium, eventually leading to the production of nanoparticles with hydroxyl groups on their surface. Raman spectroscopy was utilized to determine the kind of iron oxide in the synthetized nanoparticles. The Raman spectrum of nanoparticles is shown in Figure S1. a. The stretching vibrations of the oxygen atoms along the Fe-O bonds are related to the strongest band at 671 cm^{-1}, which is attributed to the A_{1g} mode. The T_{1g} vibrational mode is allocated to the weak bands at 321, 439, and 571 cm^{-1} [1,2]. *XRD analysis* demonstrated the crystalline nature of unmodified MNPs. Figure S1. b shows the XRD diffractogram of unmodified MNPs. The peaks at 18.4°, 30.1°, 35.5°, 36.9°, 43.2°, 53.5°, 57.2°, 62.7°, 64.5°, 71.2°, 74.4°, 79.5°, correspond to the (111), (220), (311), (222), (400), (422), (511), (440), (531), (620), (533), (622), (444) planes, correspondingly, and may be indexed utilising magnetic's face-centered cubic structure [3].

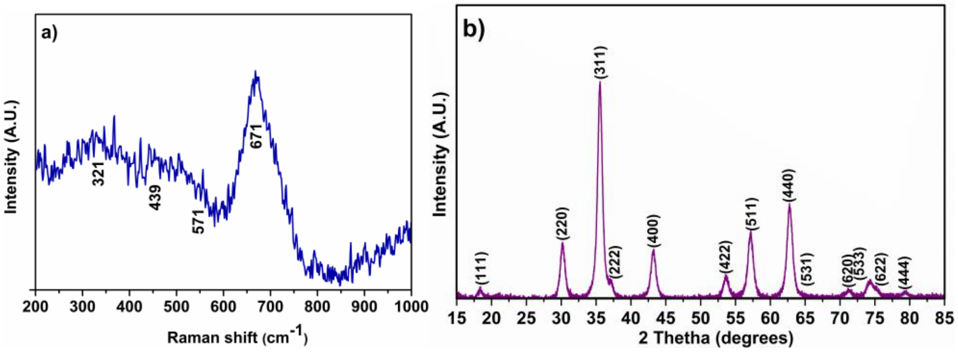

Figure S1. Characterization of MNPs synthesized with co-precipitation method: a) The Raman spectrum of the umodified MNPs, b) X-ray powder diffractogram.

Synthesis of initiator for surface-initiated atom transfer radical polymerization (SI-ATRP initiator)

Following the reaction between L-dopamine and alpha-bromobutyryl bromine, the initiator for SI-ATRP was obtained (Scheme S1).

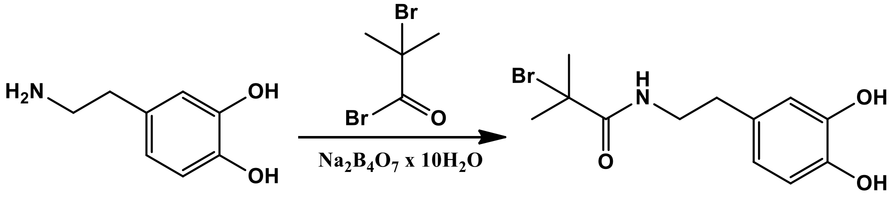

Scheme S1. Synthesis of the SI-ATRP initiator

The purity of the initiator was verified by ^{1}H-NMR, using a Bruker Avance NEO 400 MHz Spectrometer with a 5 mm inverse detection z-gradient probe, operating at 400.1 MHz for ^{1}H nucleus. The sample was dissolved in dimethyl sulfoxide-d6 (DMSO-d6) and the spectra was recorded at room temperature. Chemical shifts referred to residual solvent signal (ref. ^{1}H 2.512 ppm) and were reported in ppm.

^{1}H NMR (400 MHz, DMSO): δ 1.38 (s, 6H), δ 2.06 – 2.10 (t, 2H), δ 2.72 – 2.77 (q, 2H), δ 5.96 – 5.98 (d, 1H), δ 6.11 (s, 1H), δ 6.15 – 6.17 (d, 1H), δ 7.60 – 7.62 (t, 1H)

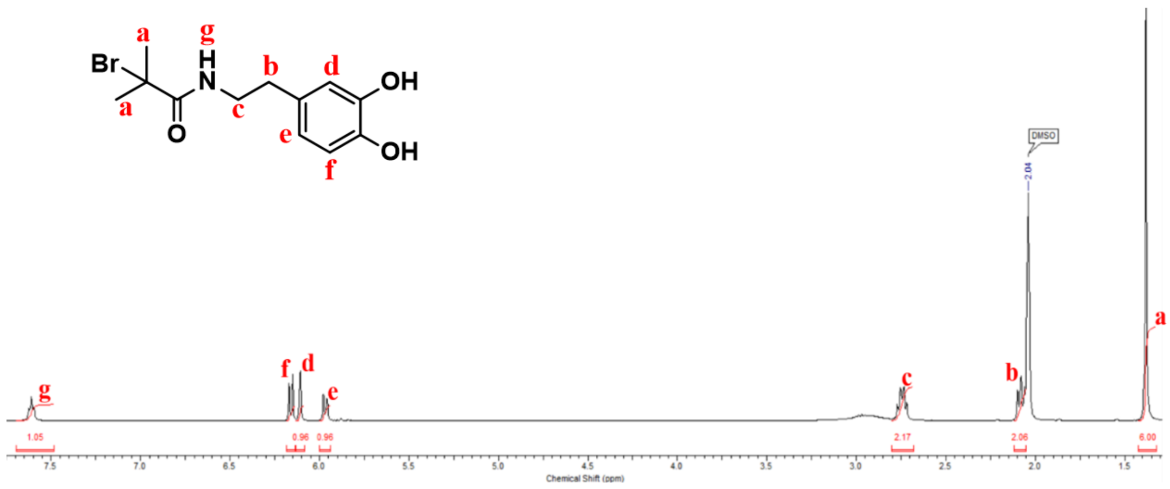

Figure S2. ^{1}H-NMR of *2-bromo-N-(3,4-dihydroxyphenethyl)-2-methylpropanamide*

Folic acid calibration curve

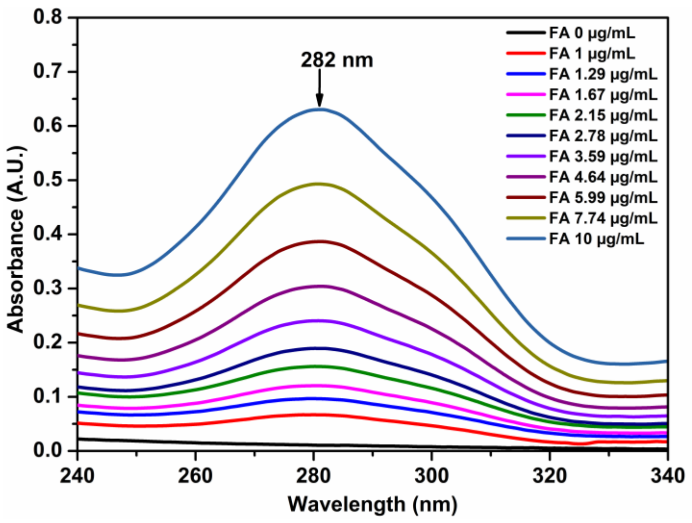

Figure S3. Folic acid calibration curve by ultraviolet-visible spectroscopy

X-ray photoelectron spectroscopy (XPS)

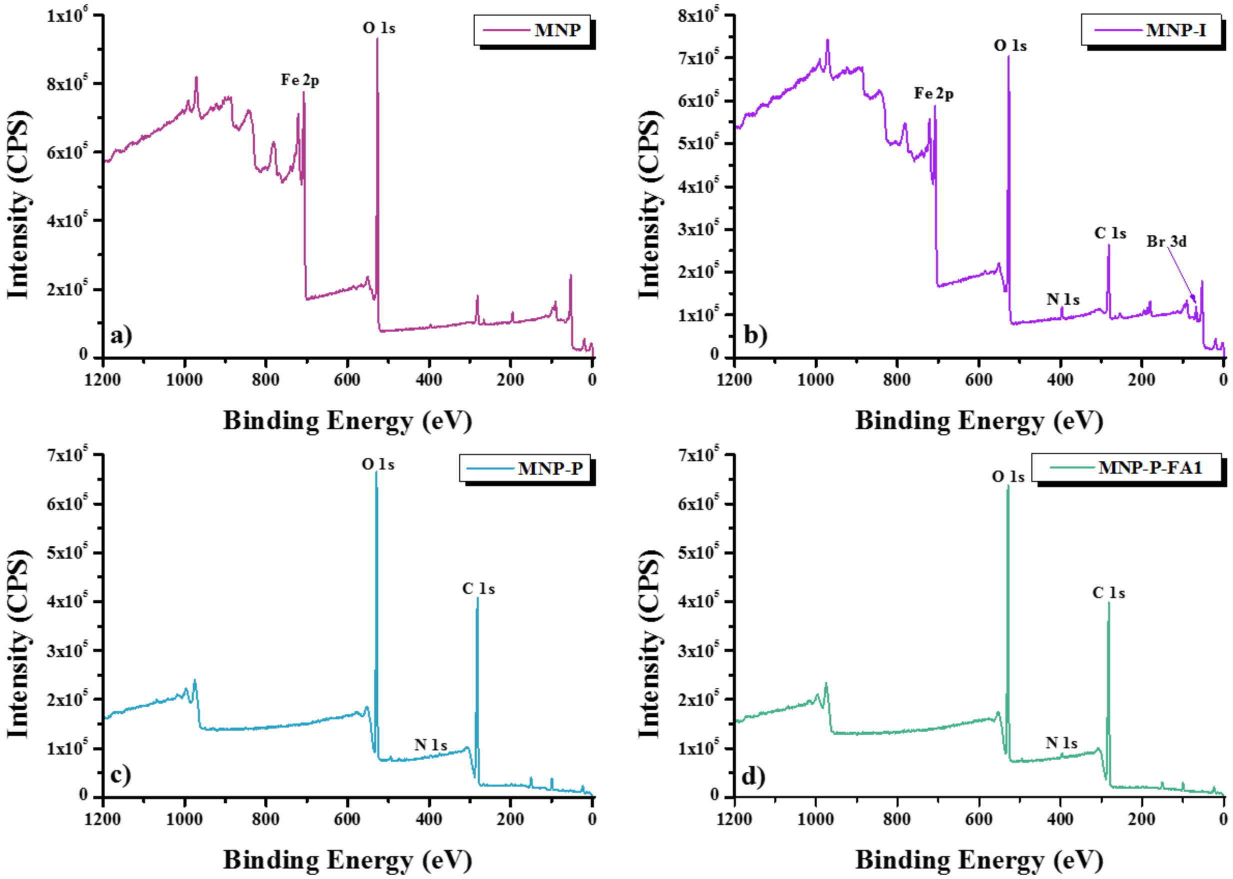

Figure S4. XPS wide scans of MNP (a), MNP-I (b), MNP-P (c), and MNP-P-FA1 (d).

Determination of the molar mass of PHEMA by thermogravimetric analysis

From TGA curve of the MNP-I sample (Figure 4) was deduced mass ratio (R_I) between iron residue and ATRP initiator of 92.16 / 5.04. From the TGA curve of MNP-P sample, the mass ratio between inorganic residue and organic material (polymer and SI-ATRP initiator) was deduced as 44.02 / 39.78. In this last case, the total mass of the analyzed sample was 11.94 mg, and by using R_I, $M_{ATRP\ intiator}$ = 302.15 g/mol and M_{HEMA}=130 g/mol a degree of polymerization = 35 was obtained.

Determination of the grafing density of the SI-ATRP initiator and of PHEMA, respectively, on the surface of the MNPs by thermogravimetric analysis

The grafting density of the SI-ATRP initiator and of the PHEMA was calculated using the adapted equation 1 from the literature [4]:

$$grafting\ density\ (d) = \frac{\left(\frac{W_{a-b\,℃}}{100-W_{a-b\,℃}}\right)\cdot 100 - W_{MNP}}{M \times S \times 100} \cdot 10^6\ (\mu mol\ /\ m^2) \quad \text{(equation 1)}$$

where: $W_{a-b\,℃}$ represents the mass loss of the organic material in the corresponding temperature range (230 - 480 °C for the SI-ATRP initiator from the MNP-I sample and 160 - 490 °C for the PHEMA from the MNP-P sample), W_{MNP} is the mass loss of organic material from the MNP sample over the temperature range of 30 – 700 °C, M is the molar mass of each compound ($M_{W_{ATRP\ initiator}} = 302.16\ /\ mol,\ M_{W_{PHEMA}} = 4550/\ mol$) and S is the surface area of MNP measured using Brunauer-Emmett-Teller adsorption (BET) with the value of 89.4 m^2/g for MNPs obtained by the co-precipitation method [5].

Dynamic light scattering (DLS) and Zeta Potential

Table S1. Dynamic light scattering and zeta potential of MNP, MNP-I, MNP-P, MNP-P-FA1 and MNP-P-FA2 in phosphate buffer 1X at pH 7.4.

Sample name	Concentration (µg/mL)	Size by DLS* (nm)	Average hydrodynamic diameter (nm ± SD*)	Zeta potential (mV)	Average zeta potential ± SD*	PDI*	Average PDI* ± SD
**MNP**	**100**	942.1	821.96 ± 121.97	-25.37	-26.12 ± 0.60	0.307	0.300 ± 0.005
		654.7		-26.15		0.295	
		869.1		-26.84		0.298	
	**50**	403.5	509.5 ± 129.14	-26.93	-26.78 ± 0.11	0.231	0.242 ± 0.009
		433.7		-26.64		0.243	
		691.3		-26.78		0.254	
	**25**	456.7	456.66 ± 5.75	-27.59	-28.47 ± 0.62	0.213	0.239 ± 0.027
		463.7		-28.90		0.229	
		449.6		-28.94		0.277	
	**12.5**	309.2	325.73 ± 17.34	-28.54	-28.09 ±	0.235	0.224 ±

		318.3		-26.78	0.94	0.215	0.008
		349.7		-28.95		0.224	
	**6.25**	317.1	311.36 ± 4.50	-28.35	-28.67 ± 0.23	0.274	0.248 ± 0.020
		306.1		-28.75		0.246	
		310.9		-28.92		0.225	
**MNP-I**	**100**	848.7	935.06 ± 62.29	-24.20	-23.77 ± 0.36	0.270	0.278 ± 0.011
		963.2		-23.82		0.271	
		993.3		-23.30		0.295	
	**50**	481.0	603.23 ± 86.45	-26.98	-26.01 ± 0.69	0.260	0.257 ± 0.002
		661.7		-25.67		0.253	
		667.0		-25.40		0.258	
	**25**	313.7	434.76 ± 93.94	-25.65	-24.77 ± 067	0.241	0.248 ± 0.005
		447.9		-24.01		0.255	
		542.7		-24.66		0.248	
	**12.5**	265.7	329.86 ± 49.27	-26.86	-27.07 ± 0.15	0.246	0.233 ± 0.011
		338.4		-27.19		0.235	
		385.5		-27.17		0.219	
	**6.25**	225.2	255.1 ± 36.84	-23.65	-24.37 ± 0.40	0.254	0.216 ± 0.027
		233.1		-24.61		0.205	
		307.0		-24.37		0.189	
**MNP-P**	**100**	1520.3	1429.7 ± 265.37	-7.91	-8.86 ± 0.89	0.325	0.316 ± 0.030
		1699.8		-8.62		0.349	
		1069.0		-10.06		0.275	
	**50**	1020.3	929.26 ± 65.50	-11.13	-10.63 ± 0.77	0.305	0.306 ± 0.009
		868.9		-9.54		0.296	
		898.6		-11.24		0.318	
	**25**	1074.8	1131.8 ± 48.61	-10.99	-10.99 ± 0.88	0.412	0.392 ± 0.013
		1127.0		-9.92		0.381	
		1193.6		-12.08		0.385	
	**12.5**	840.6	805.76 ± 55.89	-15.08	-13.09 ± 1.87	0.393	0.478 ± 0.060
		849.8		-13.62		0.530	
		726.9		-10.57		0.511	
	**6.25**	840.3	630.0 ± 153.48	-22.22	-22.38 ± 0.51	0.469	0.563 ± 0.083
		571.4		-21.85		0.549	

		478.3		-23.08		0.672	
**MNP-P-FA1**	**100**	1400.6	1173.6 ± 185.42	-24.49	-23.55 ± 1.03	0.332	0.351 ± 0.016
		946.4		-24.06		0.350	
		1173.8		-22.11		0.373	
	**50**	1360.2	1236.56 ± 88.31	-28.83	-27.61 ± 1.04	0.572	0.492 ± 0.063
		1159.4		-27.74		0.416	
		1190.1		-26.27		0.489	
	**25**	714.9	547.56 ± 122.92	-30.97	-32.00 ± 0.74	0.257	0.272 ± 0.011
		423.1		-32.35		0.286	
		504.7		-32.68		0.275	
	**12.5**	415.2	406.4 ± 23.14	-29.91	-30.12 ± 1.41	0.314	0.301 ± 0.015
		429.3		-28.51		0.280	
		374.7		-31.96		0.311	
	**6.25**	404.7	438.33 ± 38.03	-30.65	-31.86 ± 1.07	0.405	0.431 ± 0.033
		491.5		-31.67		0.411	
		418.8		-33.27		0.478	
**MNP-P-FA2**	**100**	1995.7	1996.83 ± 105.33	-32.69	-32.40 ± 0.56	0.341	0.485 ± 0.181
		1868.4		-31.61		0.374	
		2126.4		-32.91		0.742	
	**50**	1101.1	1064.46 ± 159.57	-33.81	-33.55 ± 0.21	0.847	0.827 ± 0.054
		1239.0		-33.28		0.881	
		853.3		-33.58		0.753	
	**25**	556.2	605.93 ± 52.17	-30.55	-32.36 ± 1.51	0.237	0.267 ± 0.023
		583.6		-32.28		0.270	
		678.0		-34.25		0.294	
	**12.5**	437.2	446.4 ± 13.72	-25.88	-25.99 ± 0.55	0.249	0.281 ± 0.027
		465.8		-26.72		0.280	
		436.2		-25.37		0.316	
	**6.25**	508.9	533.43 ± 24.91	-30.56	-30.54 ± 1.51	0.305	0.345 ± 0.037
		567.6		-28.68		0.336	
		523.8		-32.40		0.395	

Abbreviations: *DLS*, dynamic light scattering; SD*, standard deviation; PDI*, polydispersity index.*

Transmission electron microscopy (TEM)

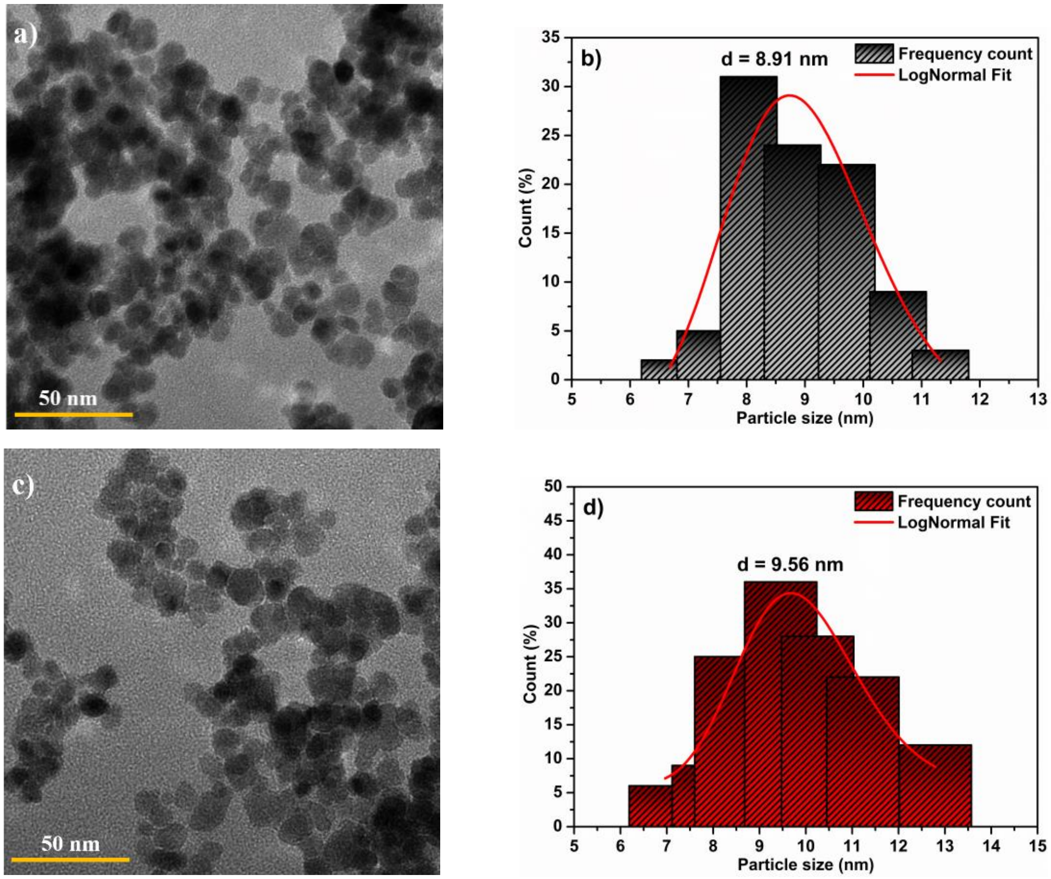

Figure S5. TEM images and histograms of particle size distribution: (a, b) MNP and (b,c) MNP-I.

Cytotoxicity assay (MTS assay)

Biocompatibility of MNP, MNP-P and MNP-P-FA was determined by MTS assay using the CellTiter 96® Aqueous One Solution Cell Proliferation Assay (Promega, Madison, WI USA), according to the manufacturer instructions. Cells were seeded into 96-well tissue culture-treated plates at a density of 1.2×10^5 cells/mL (MCF-7, HeLa, HepG2) and allowed to adhere overnight. Cells were then incubated for 24 h with fresh complete medium (control) or different sample concentrations (40, 50 and 60 µg/mL). MTS reagent was added to each well 3 h prior to absorbance readings at 490 nm on a FLUOstar® Omega microplate reader (BMG LABTECH, Ortenberg, Germany). Experiments were done in triplicate and treated cells' viability was expressed as a percentage of control cells' viability.

The nanocarriers are not cytotoxic against MCF-7, HeLa and HepG2 cancer cell lines (Figure S6).

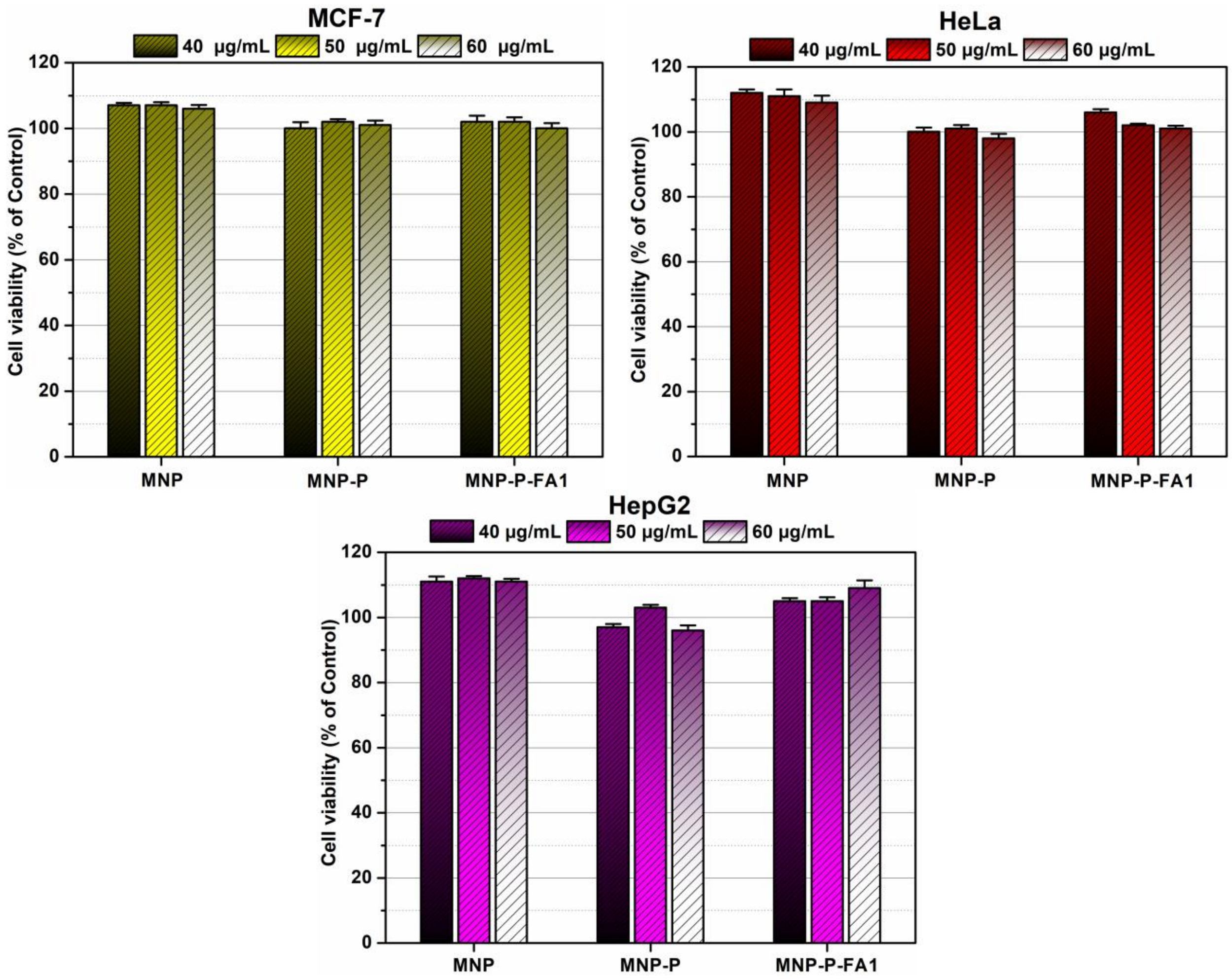

Figure S6. Biocompatibility of MNP, MNP-P and MNP-P-FA1 (40, 50 and 60 µg/mL) on breast adenocarcinoma (MCF-7), cervical adenocarcinoma (HeLa) and hepatocellular carcinoma (HepG2) cells after 24 h. Experiments were done in triplicate and treated cell viability was expressed as percentage of control cells' viability. Data were represented as means ± standard error of the mean.

References

1. Slavov, L.; Abrashev, M.V.; Merodiiska, T.; Gelev, Ch.; Vandenberghe, R.E.; Markova-Deneva, I.; Nedkov, I. Raman Spectroscopy Investigation of Magnetite Nanoparticles in Ferrofluids. *Journal of Magnetism and Magnetic Materials* **2010**, *322*, 1904–1911, doi:10.1016/j.jmmm.2010.01.005.
2. Testa-Anta, M.; Ramos-Docampo, M.A.; Comesaña-Hermo, M.; Rivas-Murias, B.; Salgueiriño, V. Raman Spectroscopy to Unravel the Magnetic Properties of Iron Oxide Nanocrystals for Bio-Related Applications. *Nanoscale Advances* **2019**, *1*, 2086–2103, doi:10.1039/C9NA00064J.
3. Schwaminger, S.; Syhr, C.; Berensmeier, S. Controlled Synthesis of Magnetic Iron Oxide Nanoparticles: Magnetite or Maghemite? *Crystals* **2020**, *10*, 214, doi:10.3390/cryst10030214.
4. Neelamegan, H.; Yang, D.K.; Lee, G.J.; Anandan, S.; Sorrentino, A.; Wu, J.J. Synthesis of Magnetite-Based Polymers as Mercury and Anion Sensors Using Single Electron Transfer-Living Radical Polymerization. *ACS Omega* **2020**, *5*, doi:10.1021/acsomega.9b03653.
5. Mascolo, M.; Pei, Y.; Ring, T. Room Temperature Co-Precipitation Synthesis of Magnetite Nanoparticles in a Large PH Window with Different Bases. *Materials* **2013**, *6*, 5549–5567, doi:10.3390/ma6125549.
